# Inhibition of cGMP‐Signalling Rescues Retinal Ganglion Cells From Axotomy‐Induced Degeneration

**DOI:** 10.1111/jnc.70072

**Published:** 2025-04-24

**Authors:** Katia Ihadadene, Azdah Hamed A Fallatah, Yu Zhu, Arianna Tolone, François Paquet‐Durand

**Affiliations:** ^1^ Graduate School INTHERAPI Burgundy University Dijon France; ^2^ Institute for Ophthalmic Research University of Tübingen Tübingen Germany; ^3^ Graduate School for Molecular Medicine University of Tübingen Tübingen Germany; ^4^ Graduate School for Cellular and Molecular Neuroscience University of Tübingen Tübingen Germany

**Keywords:** apoptosis, axotomy, diabetic retinopathy, glaucoma, Kv channels, LHON

## Abstract

The axons of retinal ganglion cells (RGCs) form the optic nerve, which relays visual information to the brain. RGC degeneration is the root cause of a variety of blinding diseases linked to optic nerve damage, including glaucoma, the second leading cause of blindness worldwide. The underlying cellular mechanisms of RGC degeneration are largely unclear; yet, they have been connected to excessive production of the signalling molecule nitric oxide (NO) by nitric oxide synthase (NOS). NO activates soluble guanylate cyclase (sGC), which subsequently produces the second messenger cyclic guanosine monophosphate (cGMP). This, in turn, activates protein kinase G (PKG), which can phosphorylate downstream protein targets. To study the role of NO/cGMP/PKG signalling in RGC degeneration, we used organotypic retinal explant cultures in which the optic nerve had been severed. We assessed the activity of NOS, RGC death and survival at different times after optic nerve transection. While NOS activity was high right after optic nerve transection, significant RGC loss occurred with a 24–48‐h delay. We then treated retinal explants with inhibitors selectively targeting either NOS, sGC, PKG, or Kv1.3 and Kv1.6 voltage‐gated potassium channels. While all four treatments reduced RGC death, the PKG inhibitor CN238 and the Kv‐channel blocker Margatoxin (MrgX) showed the most pronounced rescue effects. Our results confirm an involvement of NO/cGMP/PKG signalling in RGC degeneration, highlight the potential of PKG and Kv1‐channel targeting drugs for treatment development, and further suggest organotypic retinal explant cultures as a useful model for investigations into optic nerve damage.

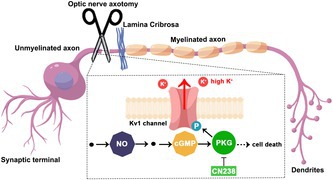

## Graphical abstract

Retinal ganglion cell (RGC) axons are myelinated only beyond the lamina cribrosa, that is, when leaving the eye. Axotomy‐induced RGC degeneration is likely to be related to excessive nitric oxide (NO) production, leading to an over‐production of cGMP, over‐activation of protein kinase G (PKG) and excessive phosphorylation of target proteins, such as voltage‐gated Kv1‐type potassium channels. The ensuing excessive outflow of K^+^ ions drains ATP supplies, eventually triggering cell death.

**Abbreviations**
7‐NI: 7‐Nitroindazole
BM: Basal medium
Ca^2+^
: Calcium
cGMP: Cyclic guanosine monophosphate
CM: Complete medium
CN238: *R*_P_‐8‐Br‐pMe‐PET‐cGMPS
DAPI: 4′,6‐diamidino‐2‐phenylindole
DMSO: Dimethyl Sulfoxide
eNOS: Endothelial nitric oxide synthase
GCL: Ganglion cell layer
INL: Inner nuclear layer
K^+^
: Potassium ion
KCNA: Potassium voltage‐gated channel subfamily A
Kv: Voltage‐gated potassium channels
MrgX: Margatoxin
NADPH: Nicotinamide adenine dinucleotide phosphate
nNOS: Neuronal nitric oxide synthase
NO: Nitric oxide
NOS: Nitric oxide synthase
NT: Non‐treated
ODQ: 1H‐ [1,2,4] Oxadiazolo [4,3‐a] quinoxalin‐1‐one
ONL: Outer nuclear layer
P: Post‐natal day
PKG: Protein kinase G
RBPMS: RNA‐binding protein with multiple splicing
RGC: Retinal ganglion cell
RPE: Retinal pigmented epithelium
SD: Standard deviation
sGC: Soluble guanylate cyclase
TUNEL: Terminal deoxynucleotidyl transferase dUTP nick end labelling
WT: Wild‐type

## 1 Introduction

Retinal ganglion cells (RGCs) are the 3rd order neurons of the retina, which collect visual information from photoreceptors and bipolar cells. RGC axons form the optic nerve and project visual information to the brain. There is a large diversity of RGCs in the retina and according to morphological, functional and molecular criteria, at least 40 different types can be distinguished (Baden et al. 2016). Diseases affecting RGCs can lead to an irreversible loss of vision and blindness. While some RGC diseases are genetic and rare, including Albinism, Leber's hereditary optic neuropathy (LHON) and autosomal dominant optic atrophy (ADOA) (Nguyen‐Ba‐Charvet and Rebsam 2020; Meyerson et al. 2015; Carelli et al. 2023), others are non‐genetic and favoured by factors like age, sex, diabetes, ocular hypertension and ethnicity. Major diseases affecting RGCs are diabetic retinopathy and glaucoma. Glaucoma is an optic nerve neuropathy resulting in progressive and irreversible blindness, affecting about 70 million people worldwide, a number that is predicted to rise to about 112 million by 2040 (Lozano et al. 2024). This makes glaucoma the second leading cause of blindness worldwide after cataract (Pereira et al. 2021). Glaucoma is characterised by a progressive loss of RGCs, with ocular hypertension as one of the main risk factors (Almasieh et al. 2012).

Numerous molecular changes have been associated with the onset of RGC degeneration, including a lack of trophic factors (Chitranshi et al. 2018) or an increased amount of reactive oxygen species (Almasieh et al. 2012). A number of studies have suggested an involvement of nitric oxide synthase (NOS) and its product nitric oxide (NO) in RGC degeneration (Neufeld 1999; Cantó et al. 2019; Mueller‐Buehl et al. 2021).

The prototypic target for NO‐signalling is the enzyme soluble guanylate cyclase (sGC), which generates the second messenger cyclic guanosine monophosphate (cGMP), using guanosine triphosphate (GTP) as substrate (Blom et al. 2012). cGMP in turn activates protein kinase G (PKG), which can phosphorylate a whole range of target proteins (Blom et al. 2012). The NO/cGMP/PKG pathway has been reported to be involved in cell death in a variety of contexts, such as in breast cancer (Fallahian et al. 2011), melanoma (Quadri et al. 2022) and cardiomyocytes (Taimor et al. 2000). Moreover, excessive cGMP/PKG‐signalling was previously shown to cause the degeneration of photoreceptor cells in the retina (Paquet‐Durand et al. 2009; Power et al. 2020). We have recently shown that inhibiting PKG can preserve the viability and functionality of RGCs in vitro (Tolone et al. 2023). While the downstream pathways triggered by PKG have not yet been elucidated, a number of PKG phosphorylation targets have been connected to retinal cell death, including voltage‐gated potassium channels belonging to the Kv1‐family (Kv1.3/KCNA3, Kv1.6/KCNA6) (Roy et al. 2022). Kv1 channels open and close in response to changes in the membrane potential, making them essential for the regulation of cell excitability (Faulkner et al. 2024). More specifically, Kv1 channels are involved in the development of RGCs and in the modulation of their electrical activity (Zhong et al. 2013). The potential involvement of Kv1 family channels in RGC degeneration was also reported in other studies, and genetic inactivation of Kv1‐channels was found to reduce RGC degeneration (Koeberle et al. 2010).

Unfortunately, there is a paucity in experimental models that faithfully reproduce the phenotypic presentation of glaucoma (Evangelho et al. 2019; Pang and Clark 2007; Schnichels et al. 2021). Nevertheless, optic nerve damage certainly leads to RGC degeneration (Munemasa and Kitaoka 2013). Therefore, to study how RGCs degenerate, we used organotypic retinal explant cultures derived from wild‐type (WT) mice in which RGCs die progressively due to the optic nerve axotomy performed during the explantation procedure (Alarautalahti et al. 2019).

Using such retinal explant cultures, we investigated the role of NO/cGMP/PKG signalling in RGC loss and included also Kv1‐channels in our studies. We employed immunofluorescence to confirm the expression of enzymes critical for this pathway, to then determine the kinetics of NOS activity, the accumulation of the NOS byproduct citrulline, RGC survival and cell death after optic nerve transection. When the results indicated that NO‐signalling likely led to RGC loss, we used various inhibitors to block either NO production, sGC‐mediated cGMP‐synthesis, PKG activity, or Kv1‐channels. These treatments markedly reduced RGC degeneration, strongly suggesting the involvement of NO/cGMP/PKG signalling and Kv1‐channel activity in RGC degeneration after optic nerve transection. Overall, this work may open up new avenues for the development of treatments of RGC diseases, possibly including glaucoma.

## 2 Material and Methods

### 2.1 Animals

**FIGURE 1:**
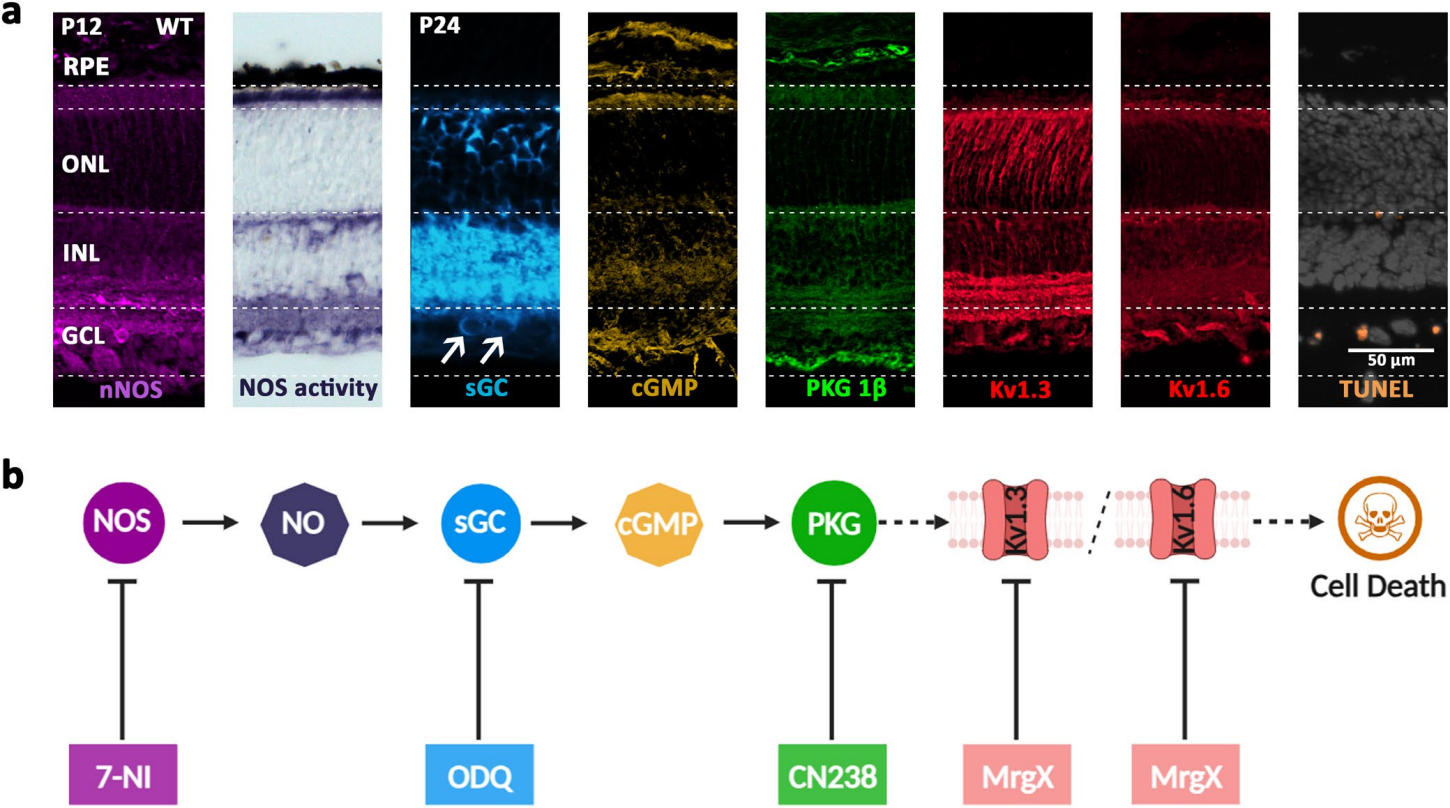
The NO/cGMP/PKG pathway in the retina, targets and inhibitors. (a) Retinal expression of enzymes and proteins involved in nitric oxide (NO)/cyclic guanosine monophosphate (cGMP) signalling. Staining performed on post‐natal (P) day 12 retina, except for sGC staining performed on P24 retina. Neuronal nitric oxide synthase (nNOS, magenta), NOS activity (purple), soluble guanylyl cyclase (sGC; cyan), cGMP accumulation (yellow), protein kinase G 1β (PKG1β; green), and Kv1.3‐ and Kv1.6‐channels (red) were all detected within the ganglion cell layer (GCL). Several of these enzymes/proteins were also expressed in the outer‐ and inner nuclear layer (ONL/INL) and the retinal pigment epithelium (RPE). The TUNEL assay (orange) was used to detect dying cells in the GCL, DAPI (grey) was used as nuclear counterstain. (b) In the prototypic NO‐signalling pathway, NOS generates NO to activate sGC and trigger cGMP production. Exceedingly high cGMP‐levels may lead to an overactivation of PKG, which phosphorylates and likely activates voltage‐gated potassium channels belonging to the Kv1 family (i.e., Kv1.3 and Kv1.6). Excessive potassium influx may trigger retinal ganglion cell (RGC) death. Different components of this pathway can be interrogated using specific inhibitors, such as 7‐nitroindazole (7‐NI) for NOS, H‐[1,2,4] oxadiazolo [4,3‐a] quinoxalin‐1‐one (ODQ) for sGC, CN238 for PKG and Margatoxin (MrgX) for Kv1.3/Kv1.6. Scale bar: 50 μm.

For all retinal explant culture experiments (see below) C57BL/6J wild‐type (WT) mice (RRID: MGI: 3028467) were used at post‐natal day (P) 12. C57BL/6J animals were purchased from Jackson Laboratories (Bar Harbor, ME, USA). Immunostaining was performed on P12 retina, except for sGC immunostaining for which P24 was used (Figure 1). Mice were housed in the specified pathogen‐free (SPF) animal facility of the Institute for Ophthalmic Research in Tübingen, in type 2 long cages, with a maximum of 5 animals per cage. The animals were housed under a standard 12/12‐h‐day and night cycle, had free access to food and water, and were used irrespective of sex (Claes and Moons 2022). Mice were sacrificed using CO_2_ asphyxiation followed by decapitation. All procedures were performed in accordance with the legislation on animal protection issued by the German Federal Government (Tierschutzgesetz) and approved by the institutional animal welfare office of the University of Tübingen under the §4 registration numbers AK02/19M and AK02/24M. All efforts were made to limit the number of animals and their suffering. The total number of animals used for this study was 99 (198 retinal explants).

### 2.2 Organotypic Retinal Explant Culture

The enucleated eyes were incubated for 5 min at room temperature (RT) in 300 μL basal R16 medium (BM) (cat. no. 07491252A; Gibco, Waltham, MA, USA). Then they were transferred to preheated BM with 0.12% (*v*/*v*) proteinase K (active) (cat. no. P6556; Sigma–Aldrich, Hamburg, Germany) and incubated for 15 min at 37°C to allow separation of the sclera and choroid from the retina and the retinal pigment epithelium (RPE). To inactivate proteinase K, the eyes were incubated for a further 5 min at room temperature in BM containing 20% (*v*/*v*) foetal calf serum (FCS) (cat. no. F7524; Sigma–Aldrich).

**FIGURE 2:**
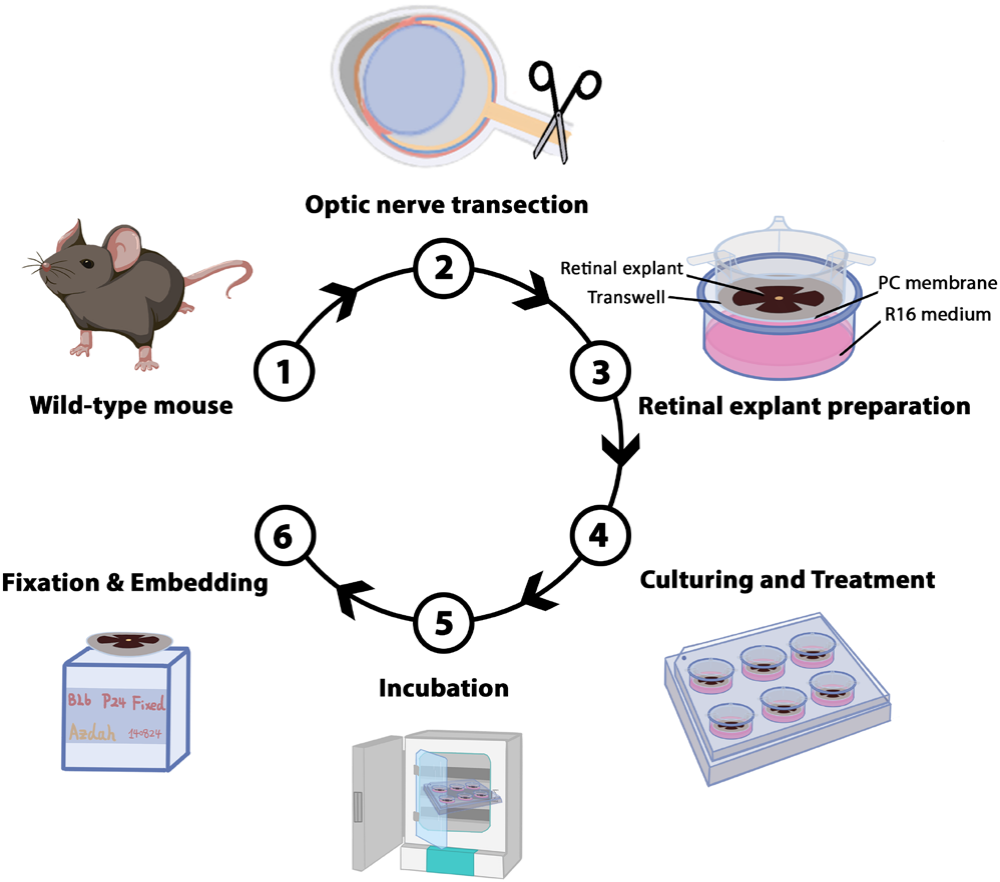
Experimental paradigm and procedures. Experiments were started with the enucleation of post‐natal day 12 (P12) wild‐type mice. Organotypic retinal explant cultures were prepared and placed on a porous polycarbonate (PC) membrane insert. They were cultured on top of a solution of complete R16 medium (CM). The retinae were incubated either in CM for different time points after optic nerve transection (0, 0.2, 0.5, 6, 12, 24 and 48 h) or treated for 24 h, 48 h or 12 days (i.e., until P24) with compounds targeting the NO/cGMP/PKG pathway. Finally, retinal explants were fixed, embedded and sectioned for subsequent staining procedures and microscopic analysis.

Afterwards, under a laminar‐flow hood and sterile conditions, the eyes were put in a petri dish containing fresh BM and dissected under the stereoscope. The retina was then cut in four points, resulting in a four‐leaf clover shape. The flattened retinae were transferred to a polycarbonate culture membrane insert (cat. no. 3412; Corning Life Sciences, Corning, NY, USA) and placed in a six‐well culture plate. 1.2 mL of complete medium (CM), prepared according to Belhadj et al. 2020, was added to the culture wells. The culture plates were placed in a humidified sterile incubator at 37°C with 5% CO_2_ (Figure 2). In longer‐term cultures (> 48 h) the CM was changed every 2 days.

To assess the involvement of NO/cGMP/PKG signalling in RGC degeneration, the following drug treatments were used: 15 μM 7‐nitroindazole (7‐NI) (cat. no. N7778; Sigma–Aldrich), 0.5 μM 1H‐[1,2,4] oxadiazolo [4,3‐a] quinoxalin‐1‐one (ODQ) (cat. no. O3636; Sigma–Aldrich), 50 μM *R*_P_‐8‐Br‐pMe‐PET‐cGMPS (CN238; cat. no. P 007; Biolog Life Science Institute GmbH & Co, Bremen, Germany), and 50 nM Margatoxin (MrgX) (cat. no. STM‐325; Alomone Labs, Jerusalem, Israel). These drugs targeted NOS, sGC, PKG, Kv1.3/Kv1.6, respectively, and were diluted in water (CN238, Margatoxin) or in dimethyl sulfoxide (DMSO; all other compounds; cat. no. 5.89569, Sigma–Aldrich) and then added to the culture medium. The maximal medium DMSO concentration was 0.1% (Tsai et al. 2009).

At the end of the culturing period, retinae were fixed in 4% (*v*/*v*) paraformaldehyde and cryoprotected by incubation in graded sucrose solutions (10%, 20%, 30%). The tissue was then embedded in OCT cryosectioning matrix (cat. no. 4583; Sakura Finetek Europe, Alphen aan den Rijn, The Netherlands) and sectioned (12 μm) using a cryotome (CryoStar NX50 OVP, Thermo Fisher Scientific, Runcorn, UK).

### 2.3 NADPH Diaphorase Assay

To examine NOS activity, retinal sections were incubated with 0.3% (*v*/*v*) TritonX100 for 2 h, then rinsed with Tris–HCL buffer (pH 8) and incubated overnight at RT with 1 mg/mL β‐nicotinamide adenine dinucleotide phosphate (NADPH; cat. no. N5130, Sigma–Aldrich) and 0.25 mg/mL nitro blue tetrazolium (NBT; cat. no. N6876, Sigma–Aldrich) dissolved in Tris–HCL. A negative control devoid of NBT was performed in parallel. Afterwards, the slides were mounted using Vectashield mounting medium for fluorescence without DAPI (cat. no. H‐1000‐10; Vector Laboratories Inc., Burlingame, CA, USA).

### 2.4 TUNEL Assay

RGC death was assessed using the Terminal deoxynucleotidyl transferase dUTP nick end labelling (TUNEL) assay (Gavrieli et al. 1992) using an in situ cell death detection kit (cat. no. 12156792910; red fluorescence; Roche, Mannheim, Germany). DAPI contained in the mounting medium (cat. no. H‐1200‐10; Vectashield antifade with DAPI; Vector Laboratories) was used as a blue fluorescence nuclear counterstain.

### 2.5 Immunostaining

Immunostaining was performed on retinal sections by incubating them with primary antibodies directed against either nNOS (1:300; Enzo Life Sciences cat. no. ALX‐210‐529‐C100, RRID: AB_2052051), sGC (1:200; cat. no. AB50288, Abcam), cGMP (1:500; a kind gift from Harry Steinbusch, Maastricht University; de Vente et al. 1987), PKG (1:100; a kind gift from Prof. Dr. Jens Schlossmann, University of Regensburg), RBPMS (1:100; OTI3B7, Novus Biologicals LLC, Centennial, CO, USA), Kv1.3 (KCNA3; 1:300, Alomone Labs cat. no. APC‐101, RRID: AB_2040149), Kv1.6 (KCNA6; 1:300, Alomone Labs cat. no. APC‐003, RRID: AB_2040158), calretinin (1:300; cat. no. MAB1568, Sigma–Aldrich), or citrulline (1:200; cat. no. 6B9207, Sigma–Aldrich). The primary antibodies were diluted in blocking solution and incubated at 4°C overnight. The sections were then incubated for 1 h at RT with secondary antibodies. These were: Goat anti‐mouse IgG (1:300; Molecular Probes cat. no. A‐11029, RRID: AB_2534088) for RBPMS and calretinin, donkey anti‐sheep (1:300; cat. no. A11015, Invitrogen, Waltham, MA, USA) for cGMP, and goat anti‐rabbit IgG (1:300; cat. no. A11034, Molecular Probes) for nNOS, citrulline, sGC, Kv1.3 and Kv1.6.

### 2.6 Microscopic Analysis and Image Processing

From each retina, six different regions were captured using a Zeiss AXIO Imager Z1 Microscope equipped with an ApoTome module, a 20x APOCHROMAT objective, and an MRm digital camera (Zeiss, Oberkochen, Germany). For the quantification of TUNEL‐positive cells and RBPMS immunostained cells, each captured image used nine *Z*‐stacks and the maximum intensity projection function. For the detection of the NADPH‐diaphorase assay, bright‐field imaging was used. Initial image processing was performed with Axiovision 4.8 software (Zeiss); final manuscript figures were assembled using Adobe Photoshop (CS5 Adobe System Incorporated, San Jose, CA, USA).

### 2.7 Cell Counting and Statistical Analysis

For the assessment of NOS activity, citrulline accumulation, amacrine cells, RGC death and survival on ex vivo specimens, for each time point, at least three biological replicates were used, each with retinae from different animals. Between 9 and 17 biological replicates were obtained for the non‐treated, control condition. The treatments with 7‐NI, ODQ, CN238 and MrgX included five biological replicates each. For the quantification, at least five pictures from each retinal explant section were captured and positively stained cells in the ganglion cell layer (GCL) were counted manually. This cell number was multiplied by the area of 1 mm^2^ (1 Mio μm^2^) and divided by the product of the length of the captured retinal section and its thickness (12 μm), to obtain the number of positive cells per mm^2^. The number of animals used was determined based on previous preliminary data. Since normality of data distribution could not be unambiguously confirmed, the experimental data were analysed using a Kruskal Wallis test and a correction for multiple comparisons using the two‐stage step up method of Benjamini, Krieger and Yekutieli (24 and 48 h treatment) as implemented in GraphPad Prism 8.0.1 (GraphPad Software Inc., La Jolla, CA, USA) or using the Mann–Whitney *U* test (1 to 1 comparison; 12‐days treatment). No pairing was used, no test for outliers was conducted, and all data obtained were included in the analysis.

## 3 Results

### 3.1 Localisation of the NO/cGMP/PKG Pathway in the RGC Layer

For an initial assessment of the NO/cGMP/PKG pathway in RGCs, we used immunofluorescence on WT mouse retina and a range of antibodies directed against neuronal NOS (nNOS), sGC, cGMP, PKG1β, Kv1.3 and Kv1.6. The inclusion of Kv1.3 and Kv1.6 in our study was motivated by previous studies that had shown an involvement of Kv1.3 in RGC degeneration (Koeberle et al. 2010). Furthermore, Kv1.3 and Kv1.6 are likely targets of PKG‐dependent phosphorylation (Roy et al. 2022), and their inhibition with Margatoxin (MrgX) affected RGC repolarisation and spiking activity (Tolone et al. 2023). Moreover, we used the NADPH‐diaphorase assay to detect NOS activity, citrulline immunostaining to assess citrulline accumulation, and the TUNEL assay to identify dying cells in situ, in RGCs (Figure 1). This opening study showed that all the components of NO/cGMP/PKG‐signalling, including nNOS, sGC, PKG1β, Kv1.3 and Kv1.6, were expressed in murine RGCs, and that NOS activity, cGMP and cell death could be readily detected in RGCs.

### 3.2 NOS Activity Is High After Optic Nerve Transection

**FIGURE 3:**
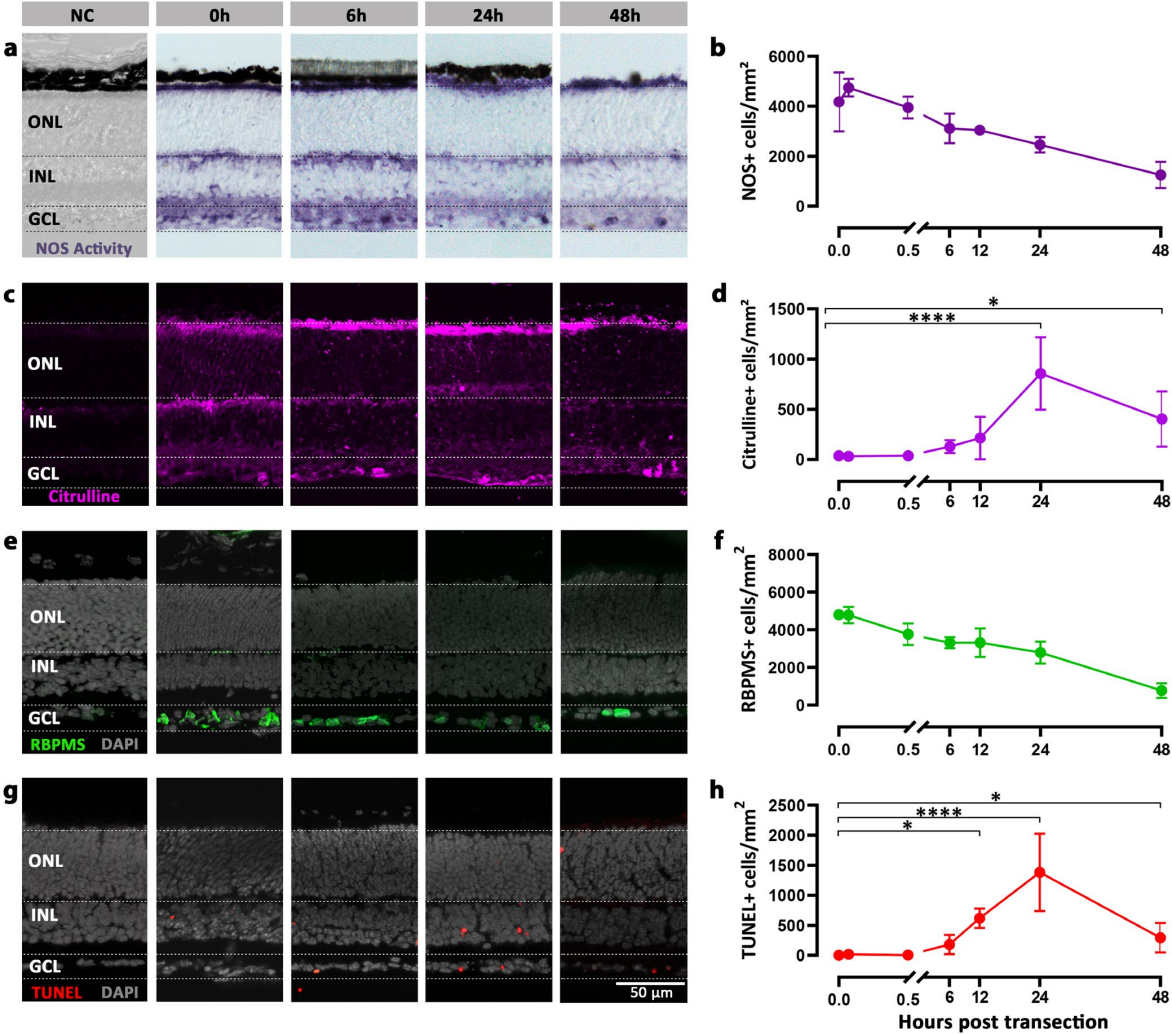
NOS activity after optic nerve transection correlates with RGC death. Organotypic retinal explant cultures derived from wild‐type (WT) mice at post‐natal day 12 were cultured for different time points after optic nerve transection (0, 0.2, 0.5, 6, 12, 24, 48 h). (a, b) NADPH diaphorase assay (bright‐field) detected NOS activity (purple) in the ganglion cell layer (GCL). Remarkably, NOS activity was already high at the first analysis time‐point after optic nerve transection and decreased gradually over time. (c, d) Citrulline immunostaining (magenta) detects citrulline accumulation in the GCL. (e, f) RBPMS immunostaining (green) detected retinal ganglion cells (RGCs). Their numbers steadily decreased after axotomy. (g, h) TUNEL assay (red) detected dying RGCs. Cell death peaked at 24 h after optic nerve transection. Quantifications indicate positive cells/mm^2^; data points in line graphs represent retinal explants from different animals; testing was performed on *n* = 3–5 animals using the non‐parametric Kruskal–Wallis test followed by the two‐stage step‐up method of Benjamini, Krieger and Yekutieli as post hoc test; significance levels: *p ≤ 0.05, ****p ≤ 0.0001. Error bars: mean with SD. INL, inner nuclear layer; NC, negative control; ONL, outer nuclear layer; scale bar: 50 μm.

To assess the kinetics of NOS activity during the first two days after optic nerve transection, we used WT mouse retina explanted at P12 and compiled a time‐series for NOS activity, citrulline accumulation, that is, a byproduct of NOS activity, as well as RGC cell death and survival. The results obtained indicated elevated NOS activity (NOS activity‐positive GCL cells: 4173 ± 1183 SD) already at the earliest time points after optic nerve transection. Citrulline‐positive cells were almost undetectable (Figure 3a,b), while cell death in the retinal GCL, as assessed by the TUNEL assay, was initially very low (TUNEL‐positive cells: 16.3 ± 14.2 SD) (Figure 3e,f). At the earliest post‐axotomy time points, the normal, full complement of RBPMS‐positive cells was detected, averaging 4801 (±225 SD) cells/mm^2^ (Figure 3c,d), a value that agrees well with previous data for the mouse retina (Dräger and Olsen 1981). During the first 12 h after axotomy, the numbers of RGCs showing NOS activity remained high, while the numbers of citrulline‐ and TUNEL‐positive cells started to rise. At the same time, the overall number of RBPMS‐positive cells decreased. At 24 h post optic nerve transection, NOS activity and RBPMS‐positive cells decreased significantly (NOS activity positive GCL cells: 2457 ± 303 SD; RBPMS positive cells: 2787 ± 585 SD) whereas the number of citruslline‐ and TUNEL‐positive cells reached a peak (citrulline‐positive cells: 856 ± 361, *p*: 0.0006; TUNEL‐positive cells: 1882 ± 643 SD, *p*: 0.0006). Finally, at 48 h, NOS activity, as well as citrulline‐, RBPMS‐ and TUNEL‐positive cells, were strongly decreased. At this time point, around 85% of the RBPMS‐positive RGCs were lost, with only 774 (±387 SD) cells/mm^2^ remaining (Figure S2). These data suggest that RGC axotomy produced an immediate effect, characterised by a rise in NOS activity and a rapid loss of RGCs, typical of an acute condition.

### 3.3 Inhibition of NOS, sGC, PKG and Kv1 Prevents Optic Nerve Transection‐Induced RGC Death

To confirm the involvement of the NO/cGMP/PKG pathway in RGC degeneration, organotypic retinal explant cultures were treated separately with inhibitors targeting NOS, sGC and PKG, as well as Kv1.3 and Kv1.6, for a duration of either 24 or 48 h. The drugs used to this effect were 7‐nitroindazole (7‐NI), 1H‐[1,2,4]oxadiazolo[4,3,‐a]quinoxaline‐1‐one (ODQ), *R*_P_‐8‐Br‐pMe‐PET‐cGMPS (CN238) and Margatoxin (MrgX), respectively. MrgX was originally identified in venom extracts obtained from the scorpion *Centruroides margaritatus* and shown to be highly specific for Kv1.3 and Kv1.6 (Garcia‐Calvo et al. 1993). Non‐treated (NT) retinae, maintained in regular CM, were kept as control. Because some compounds were diluted in 0.1% (*v*/*v*) DMSO, retinal explants were also treated with this solvent as an additional negative control. A previous study (Tsai et al. 2009) had suggested that a concentration exceeding 0.1% may be toxic for retinal cells. Yet, in our hands, 0.1% DMSO treatment had no effect on the retina, and hence, for subsequent statistical analysis, the data from DMSO treated and NT conditions were combined (Figure S1).

**FIGURE 4:**
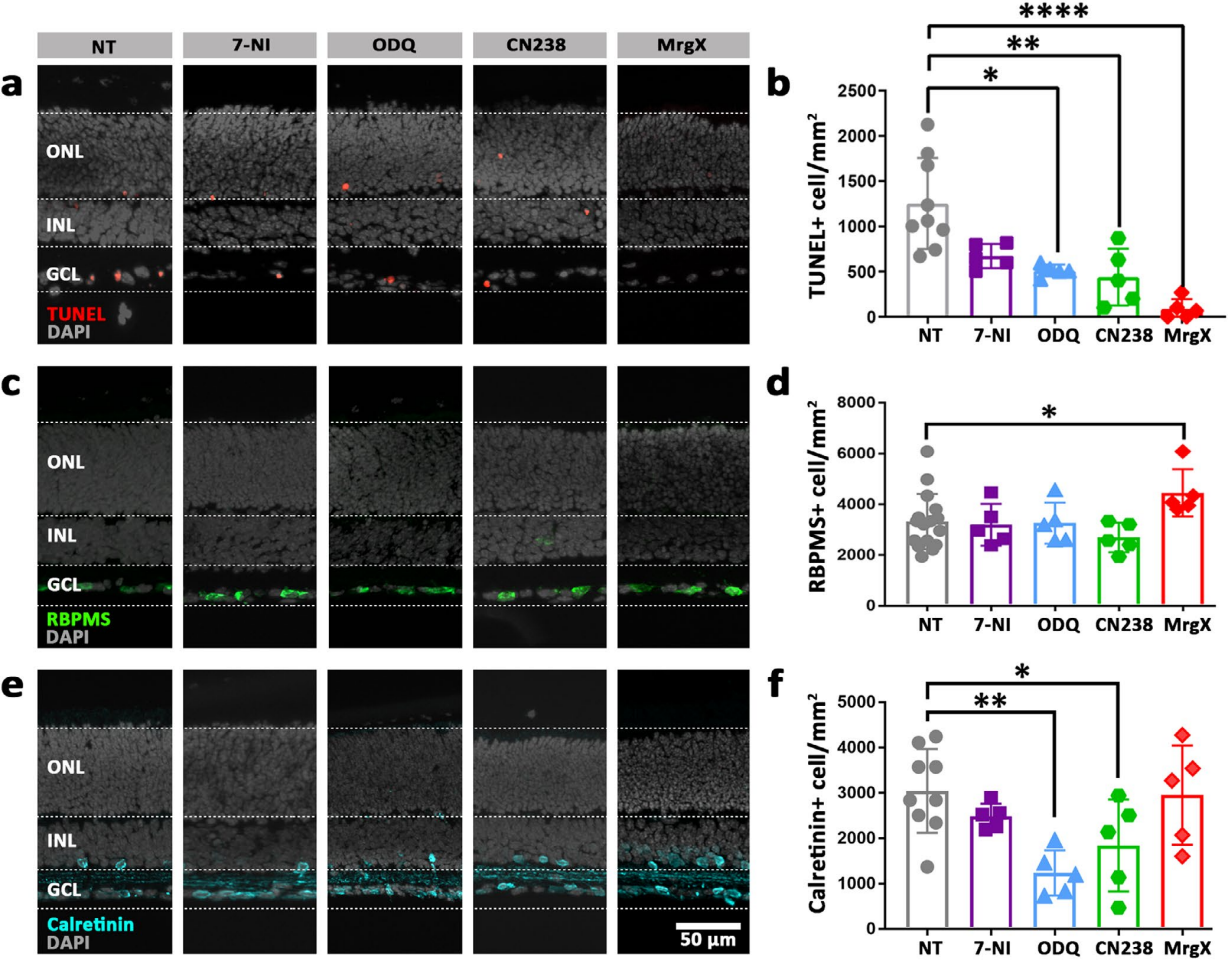
Short‐term NO/cGMP/PKG pathway inhibition reduces RGC degeneration. Retinal explant cultures derived from wild‐type (WT) mice at post‐natal day 12 were treated for 24 h with 15 μM 7‐NI, 0.5 μM ODQ, 50 μM CN238 or 50 nM MrgX; that is, inhibitors targeting NOS, sGC, PKG, Kv1.3 and Kv1.6, respectively. Non‐treated (NT) retina served as control; DAPI (grey) was used as nuclear counterstain. (a, b) TUNEL assay (red) and quantification of TUNEL‐positive cells within the ganglion cell layer (GCL). A significant reduction of TUNEL‐positive cells was noticed with all inhibiting drugs, except with 7‐NI. (c, d) RBPMS‐immunostaining (green) and counts of RBPMS‐positive retinal ganglion cells (RGCs). MrgX significantly preserved RGCs. (e, f) Calretinin‐immunostaining (cyan) and counts of calretinin‐positive amacrine cells in the GCL. ODQ and CN238 treatments appeared to reduce GCL amacrine cell numbers. Quantifications indicate positive cells/mm^2^; data points in bar graphs represent retinal explants from different animals; testing was performed on *n* = 5–16 animals. Error bars: mean with SD; treatments were compared to NT using the non‐parametric Kruskal–Wallis test followed by the two‐stage step‐up method of Benjamini, Krieger and Yekutieli as post hoc test; significance levels: *p ≤ 0.05, **p ≤ 0.01, ****p ≤ 0.0001. INL, inner nuclear layer; ONL, outer nuclear layer; scale bar: 50 μm.

At 24 h, all four drug treatments reduced the numbers of dying RGCs, as assessed by the TUNEL assay. While this decrease did not attain statistical significance for the 7‐NI treatment, a statistically significant reduction was observed with ODQ, CN238 and MrgX treatment, confirming the involvement of the NO/cGMP/PKG and Kv1 pathway in axotomy‐induced RGC degeneration (Figure 4a,b). At 24 h, the RBPMS immunostaining did not show treatment‐dependent changes in the number of surviving RBPMS‐positive cells, except for MrgX (Figure 4c,d). With a value of 4448 (±931 SD) the number of remaining RBPMS‐positive cells in the 24 h cultured, MrgX treated retina compared favourably to the initial 0 h situation (4801 ± 225 SD cells/mm^2^; see above).

As a further control for possible toxic effects of these treatments on cells in the ganglion cell layer (GCL), we used calretinin staining, that is, a label for amacrine cells, located both at the inner border of the inner nuclear layer (INL) and within the GCL. Quantification of calretinin‐positive cells within the GCL revealed a significant decrease after 24 h treatment with ODQ and CN238 (*p*: 0.0018, 0.0488, respectively), while 7‐NI and MrgX had no effect (*p*: 0.4167, 0.7914, respectively).

**FIGURE 5:**
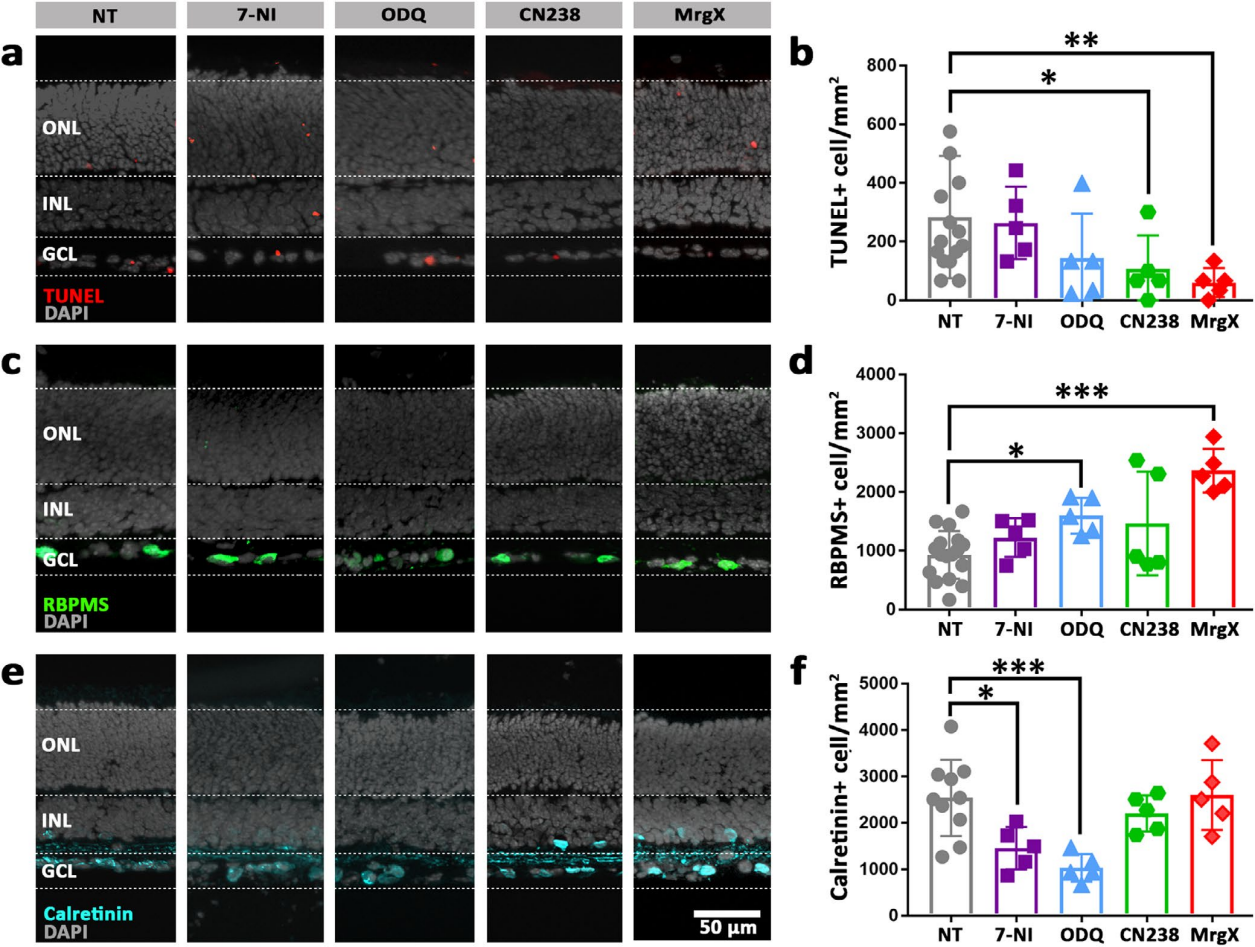
Forty‐eight‐hour inhibition of NO/cGMP/PKG‐signalling preserves RGC viability. Retinal explant cultures derived from wild‐type (WT) mice at post‐natal day 12 were treated for 48 h with 15 μM 7‐NI, 0.5 μM ODQ, 50 μM CN238, or 50 nM MrgX, targeting NOS, sGC, PKG, Kv1.3 and Kv1.6, respectively. Non‐treated (NT) retina served as control; DAPI (grey) was used as nuclear counterstain. (a, b) TUNEL assay (red) and quantification of TUNEL‐positive cells within the ganglion cell layer (GCL). A significant reduction of TUNEL‐positive cells was noticed with CN238 and MrgX. (c, d) RBPMS‐immunostaining (green) and counts of RBPMS‐positive retinal ganglion cells (RGCs). ODQ and MrgX significantly preserved RGCs. (e, f) Calretinin‐immunostaining (cyan) and counts of calretinin‐positive amacrine cells in the GCL. 7‐NI and ODQ treatments markedly reduced GCL amacrine cell count. Quantifications indicate positive cells/mm^2^; data points in bar graphs represent retinal explants from different animals; testing was performed on *n* = 5–17 animals. Error bars: mean with SD; treatments were compared to NT using the non‐parametric Kruskal–Wallis test followed by the two‐stage step‐up method of Benjamini, Krieger and Yekutieli as post hoc test; significance levels: *p ≤ 0.05, **p ≤ 0.01, ***p ≤ 0.001. INL, inner nuclear layer; ONL, outer nuclear layer.

After this initial assessment of drug effects on RGC degeneration, we prolonged the treatment duration to 48 h to test whether RGC preservation could be sustained over a longer period of time. After 48 h of treatment, NOS and sGC inhibitors had no significant effect on the RGC death rate, while PKG and Kv1 channel inhibition significantly reduced the numbers of TUNEL‐positive RGCs (Figure 5a,b). The number of surviving RBPMS‐positive cells was significantly higher after inhibition of sGC and Kv1 channels; yet, no significant difference was observed with NOS and PKG inhibitors (Figure 5c,d). Calretinin staining reported decreased numbers of GCL amacrine cells after 48 h treatment with NOS and sGC inhibitors, while CN238 and MrgX had no effect on amacrine cell viability (Figure 5e,f).

### 3.4 Inhibition of PKG Rescued RGCs After 12 Days of Culture

The results obtained in the 24‐ and 48‐h long treatments confirmed an involvement of the NO/cGMP/PKG pathway in RGC degeneration and highlighted this pathway for possible therapeutic interventions. However, the compounds 7‐NI and ODQ displayed significant negative effects on amacrine cell viability, disqualifying these compounds for longer‐term administration. Similarly, the marked (neuro)toxicity of MrgX and related scorpion venoms targeting Kv1 channels (Brenes and Gómez 2016) may preclude applications beyond basic research. We therefore chose to continue with further testing on long‐term retinal explant cultures with the PKG inhibitor CN238.

**FIGURE 6:**
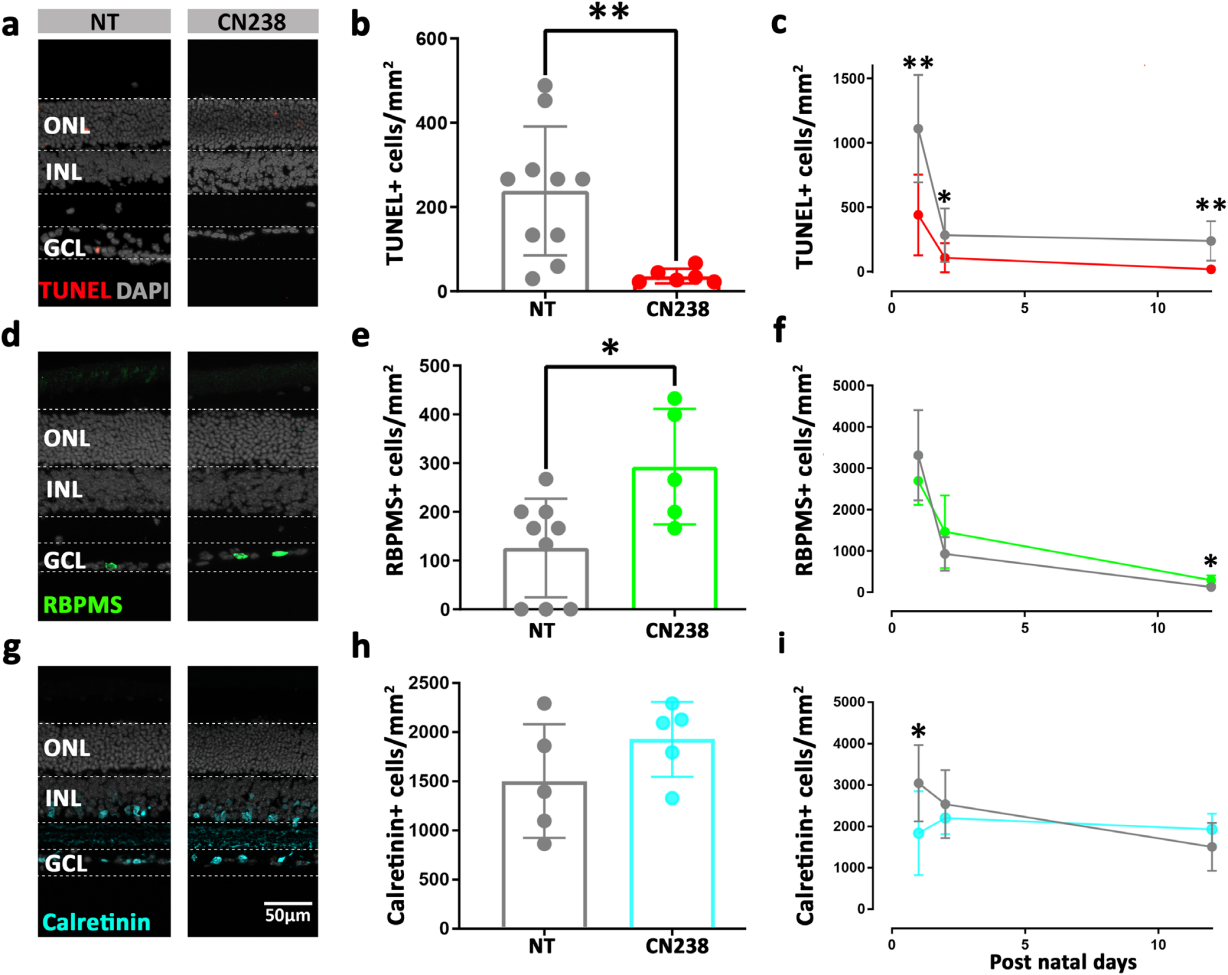
Treatment with CN238 preserves retinal ganglion cells in the long‐term. Retinal explant cultures derived from wild‐type (WT) mice at post‐natal day 12 were treated for a further 12 days with 50 μM CN238. (a, b) TUNEL assay (red) and quantification of TUNEL‐positive cells within the ganglion cell layer (GCL). (c) Compared to non‐treated (NT) controls, CN238 produced a significant and consistent reduction of TUNEL‐positive cells over 12 days of treatment. (d, e) RBPMS‐immunostaining (green) and counts of RBPMS‐positive retinal ganglion cells (RGCs). (f) CN238 significantly preserved RGCs. (g, h) Calretinin‐immunostaining (cyan) and counts of calretinin‐positive amacrine cells in the GCL. (i) Amacrine cell counts were essentially unchanged by CN238 during the 12 treatment days. Quantifications indicate positive cells/mm^2^; data points in bar graphs represent retinal explants from different animals; testing was performed on *n* = 5–10 animals. Error bars: mean with SD; treatments were compared to NT using the non‐parametric Mann–Whitney *U* test for 12‐day treatment and the non‐parametric Kruskal–Wallis test followed by the two‐stage step‐up method of Benjamini, Krieger and Yekutieli as post hoc test for 24 and 48 h treatments; significance levels: *p ≤ 0.05, **p ≤ 0.01, ***p ≤ 0.001. INL, Inner nuclear layer; ONL, Outer nuclear layer; scale bar: 50 μm.

Long‐term treatment of retinal explants for 12 days in vitro with CN238 showed preservation of RGCs as indicated by a strong decrease of TUNEL‐positive cells (Figure 6a,b). An overview of the progression of cell death during the entire treatment period indicated a general delay in RGC degeneration at 1, 2 and 12 days of CN238 treatment (Figure 6c). Moreover, 12 days of treatment resulted in an almost 3‐fold increase in the numbers of RBPMS‐positive cells (Figure 6d,e) and an overall increased survival of RGCs (Figure 6f). Finally, over the 12‐day treatment period the number of calretinin‐positive amacrine cells remained essentially stable at values between 2000 to 3000 cells/mm^2^, in line with previous literature (Jeon et al. 1998; Costa et al. 2020) and with no major differences between CN238‐treated and NT retina (Figure 6g–i).

## 4 Discussion

Previous studies have related RGC degeneration to the activity of NO, often focusing on its direct protein‐ and lipid nitrosylating effect (Cheung et al. 2000; Koriyama et al. 2011). In the present work, we investigated the effects that NO exerted via cGMP and PKG signalling. Notably, we found that inhibition of the NO/cGMP/PKG pathway had a marked protective effect on axotomised RGCs. While we cannot rule out the possibility that RGC protection was mediated by drug effects on other retinal cell types (e.g., bipolar cells, Müller glia cells), our study highlights the importance of this pathway for RGC viability and may open up new perspectives for the treatment of glaucoma and other retinal diseases caused by RGC degeneration.

## 5 NO Involvement in RGC Degeneration

The gaseous signalling molecule NO mediates cellular and transcellular communication and a diverse set of functions, ranging from smooth muscle relaxation to immune response to synaptic plasticity, neurite outgrowth and axonogenesis (Ignarro 1990; Bicker 2005). The NO‐synthesising enzyme NOS is activated by elevated Ca^2+^ (Andrew and Mayer 1999), explaining why after RGC axotomy NOS activity increased strongly and almost immediately. While NO can act on many proteins (and lipids) via S‐nitrosylation, a key effector of NO‐signalling is sGC, which strongly amplifies the response to NO stimulation via production of the second messenger cGMP (Garthwaite 2005). Our data show the functional expression of the key components of the NOS/NO/sGC/cGMP/PKG pathway in mouse RGCs, in line with earlier data obtained on Ferret (Leamey et al. 2001) and Salamander retina (Blom et al. 2009).

Especially during the 1990s and 2000s, cGMP‐signalling was often highlighted as neuroprotective (Wang and Robinson 1997; Fiscus 2002; Pilz and Broderick 2005) and this concept triggered major drug development programmes, including at the German pharmaceutical company Bayer (Sandner et al. 2021). This happened in spite of strong experimental evidence, available at the time, showing that cGMP had either no neuroprotective effects or that high levels of cGMP exacerbated neurodegenerative conditions (Oppenheim 1985; Dawson et al. 1991; Li et al. 1997; Montoliu et al. 1999). In the context of retinal degenerative diseases, conclusive evidence for the destructive effects of cGMP has been published as far back as the early 1970s (Farber and Lolley 1974; Lolley et al. 1977).

In RGCs, multiple lines of evidence indicate the detrimental effects of NO. In mice in which the neuronal isoform of NOS (nNOS) had been knockedout, RGCs were found to be resistant to NMDA‐induced excitotoxic damage. However, the knock‐out of the endothelial NOS isoform (eNOS) had no effect on NMDA‐dependent RGC degeneration (Vorwerk et al. 1997). Moreover, NOS activity was found to promote RGC degeneration in cultured rat retinal explants in vitro (Katsuki et al. 2004) and in rats in vivo after hypoxia (Rathnasamy et al. 2014). A more recent transcriptomic study on human post‐mortem retinal samples derived from patients with diabetic retinopathy suggested an increased activation of the cGMP/PKG‐signalling pathway during disease progression (Becker et al. 2021). In our study, a large fraction of RGCs stained positively in the NADPH‐diaphorase assay for NOS activity, even at the earliest time points post axotomy. Furthermore, the progression of NOS activity was correlated to the loss of RGCs in the 48 h after retinal explantation. In addition, an accumulation of citrulline, a NOS activity precursor and byproduct, was noticed with a peak at 24 h followed by a gradual decrease at 48 h indicating an inefficient citrulline metabolism in the dying RGCs leading to the decrease in the NOS activity observed (Baydoun et al. 1994). Together, this indicated a rapid activation and potential involvement of NO‐signalling in RGC degeneration.

Because of these antecedents and the known detrimental effects of cGMP in retinal photoreceptors (Power et al. 2020), we raised the hypothesis that not only direct NO actions via protein S‐nitrosylation but the entire NO/cGMP/PKG amplification cascade might be involved in RGC degeneration induced by axonal damage.

## 6 PKG Activity Likely Promotes RGC Degeneration

In retinal photoreceptors, the destructive effect of cGMP‐dependent PKG activation is well established (Paquet‐Durand et al. 2009; Xu et al. 2013; Yang et al. 2020) For RGCs, the destructive role of excessive NO‐signalling is also rather clear (Neufeld 1999; Rathnasamy et al. 2014); yet, the role of cGMP and PKG in RGC degeneration is less obvious. Previous studies found PKG to be expressed in salamander and mouse RGCs (Blom et al. 2009, 2012), in line with our data. In salamander RGCs, NO was shown to cause a cGMP and PKG‐dependent increase in intracellular Ca^2+^‐levels (Hirooka et al. 2000), demonstrating the functionality of the NO/cGMP/PKG pathway in RGCs.

In micro‐electrode‐array (MEA) recordings performed on acute murine retinal explants, the PKG activator CN052 produced a high RGC spiking activity and decreased the time RGCs needed for membrane repolarisation after KCl‐induced depolarisation (Tolone et al. 2023). The PKG inhibitor CN238 produced the exact opposite effects on RGC spiking activity and membrane repolarisation, an effect that was very similar to what was seen with MrgX treatment. Since Kv1.3 and Kv1.6 are likely PKG targets (Roy et al. 2022), this finding suggests that their PKG‐dependent phosphorylation can modulate overall RGC activity.

To test for the involvement of PKG in RGC degeneration, we used pharmacological inhibitors to block either PKG or the upstream activator sGC. Inhibition of both enzymes resulted in marked protection of RGCs, strongly suggesting a detrimental role for PKG in RGC pathogenesis. At this point it was, however, still unclear how PKG activity could precipitate RGC death.

## 7 Energy Expenditure, Kv1 Channels and RGC Death

The degenerative downstream pathways triggered by PKG activation are still only poorly understood and, notably, it is not clear what protein targets may be phosphorylated by PKG and what consequence such a phosphorylation would have. In a recent study (Roy et al. 2022) several possible PKG substrates in the retina were identified, including voltage potassium channels belonging to the Kv1 family (KCNA3, KCNA6). In our study, we focused on Kv1.3 and Kv1.6 channels since these PKG targets were known to be localised to RGCs (Höltje et al. 2007). Inhibiting Kv1.3 and Kv1.6 appeared to have a positive impact on RGCs, in line with earlier studies on the role of these channels in neurodegenerative diseases, such as multiple sclerosis (Judge et al. 2006). More specifically, in mouse retina, blocking Kv1 channels in vivo was reported to rescue RGCs (Koeberle and Schlichter 2010).

Why would (excessive) Kv1 channel activity be detrimental for RGCs? Under physiological conditions, the K^+^‐efflux mediated by Kv1 channels is important to allow for the rapid repolarisation of the axonal membrane after an action potential (Zhong et al. 2013). PKG‐dependent phosphorylation of Kv1 channels likely increases their activity and thus K^+^‐efflux (Tolone et al. 2023). This K^+^‐efflux must be compensated for by the ATP‐driven Na^+^/K^+^‐eXchanger (NKX), an ATPase, which in most neurons is responsible for up to 50% of total ATP expenditure (Pivovarov et al. 2018). In RGCs, whose metabolism may be already compromised by axonal damage, an overall greater energy expenditure is highly likely to precipitate cell death. Conversely, a reduction of Kv1 channel activity by either direct inhibition with, for instance, MrgX, or an indirect modulation via PKG inhibition, may reduce the strain on RGC energy metabolism, allowing the cell to focus its efforts on preservation rather than membrane potential maintenance. In this context, it also appears that the organotypic retinal explant culture system may be particularly well suited to study and manipulate degenerative processes triggered by axonal damage.

## 8 Organotypic Retinal Explant Culture as a Model for Studies Into RGC Degeneration

In diseases affecting RGCs, including in glaucoma, the main characteristic is a progressive loss of RGCs and their axons, leading to a loss of the visual field (Jayaram et al. 2023). There are various cellular and molecular changes that contribute to the degeneration of RGCs, including damage to the optic nerve, an elevation of the intraocular pressure, inflammation, genetic mutations, ischaemia and lack of trophic factors (Baudouin et al. 2021; Pang and Clark 2007; Almasieh et al. 2012). This complexity and the incomplete understanding of the pathogenesis of RGC degeneration require careful consideration for the choice of appropriate disease models. Notably, it is still a significant challenge to mimic disease‐associated alterations in experimental models (Evangelho et al. 2019).

However, one factor that is undoubtedly connected to glaucoma and other RGC degenerative diseases is axonal damage (Nuschke et al. 2015). In this sense, the optic nerve transection performed during retinal explantation and the progressive loss of RGCs during subsequent culture reproduces a key aspect of RGC degenerative diseases. Significantly, retinal organ culture preserves the structural integrity of the retina and crucial cell‐to‐cell interactions that are important for RGC maintenance. In a previous study using retinal explant cultures derived from adult rats, a variety of different compounds, including brain‐derived neurotrophic factor (BDNF), were tested for their neuroprotective efficacy (Bull et al. 2011). A similar culture system, using murine retinal explants, was employed to measure light‐evoked RGC activity on a MEA recording system over 14 days of in vitro culture (Alarautalahti et al. 2019). Deppe and collaborators have used porcine retinal explants to investigate the protective effect of Coenzyme Q10 against oxidative stress that plays a role in neurodegenerative diseases such as glaucoma (Deppe et al. 2024). Mueller‐Buehl et al. 2021, have used the same model to study the effect of oxidative stress on autophagy activation in the context of glaucoma.

The mouse retinal explant culture system used in this study was developed already in the late 1980s (Caffé et al. 1989) and continuously refined over the last decades (Belhadj et al. 2020; Tolone et al. 2023). As opposed to many other retinal explant procedures, our study used retinal explants cultivated together with their native RPE cell layer, in serum free medium, without antibiotics, so as to allow for targeted manipulations under entirely controlled conditions. In this retinal culture system, we observed an initial rapid loss of RGCs during the first 2 days of culture, with a slower progression of degeneration over the next 10 days in culture. At this point in time, the number of surviving RGCs had dropped to a few hundred cells per mm^2^, in agreement with earlier studies (Alarautalahti et al. 2019). Overall, this culture system allowed us to follow the progression of RGC degeneration and to assess the underlying biochemical processes with cellular resolution, highlighting its usefulness for further studies into RGC pathogenesis and neuroprotection.

However, our findings are based on an acute RGC degeneration model and have shown that the inhibitors are effective in this context. The latter findings will require further validation in more chronic models. Nevertheless, our results showing strong RGC losses align with what has been observed in humans. Indeed, Medeiros et al. (2013) have reported that the estimated average RGC count in eyes with early visual field defects was 652 057 ± 115 829 cells, which was significantly lower than the 910 584 ± 142 412 cells observed in healthy eyes (*p*: < 0.001). This corresponds to an average RGC loss of 28.4% in glaucoma‐affected eyes, with individual losses ranging from 6% to 57% at the onset of the earliest visual field defect detected by standard automated perimetry.

This ex vivo model offers valuable advantages compared to in vivo models, as it satisfies an important criterion regarding animal ethics: The reduction in the number of animals used in experiments. In addition, it allows for the design of multiple experimental conditions and, consequently, the study of different molecular signalling pathways using various pharmacological and genetic tools in both physiological and disease contexts in animal and post‐mortem retina. It also enables the investigation of early, cell‐specific death mechanisms. However, like any experimental model, it has limitations that should be taken into consideration. Retinal explants are devoid of blood circulation; therefore, the potential contribution of circulating factors, including adaptive immunity, cannot be evaluated (Valdés et al. 2016). The processes that can be investigated are also limited by time, as the cells progressively die.

In the present work, immunohistochemical analyses were performed to highlight the involvement of the NO/cGMP/PKG signalling pathway in RGC loss, which occurs in many ocular diseases. These data support previously obtained results regarding the lethal role that PKG may play in retinal cells (Paquet‐Durand et al. 2009; Tolone et al. 2023). Further studies using different experimental techniques are needed to confirm these observations for RGC degeneration.

## 9 Conclusion

In the present study, we found NOS activity to exacerbate RGC degeneration via an increased activation of the NO/cGMP/PKG signalling pathway. We show that Kv1‐type channels, likely downstream targets for PKG, further precipitate RGC death. The involvement of Kv1‐type channels in the degenerative process is highly interesting from a mechanistic point of view as it may suggest that RGC degeneration ultimately is linked to increased energy expenditure brought about by the necessity to maintain axonal membrane potentials. Moreover, we show that inhibition of the NO/cGMP/PKG pathway at any stage results in some form of RGC protection, highlighting this pathway as a target for therapeutic intervention. This ex vivo model may contribute to further advances in the understanding of the molecular and cellular mechanisms underlying this pathology and to new indications for potential therapeutic targets.

## Author Contributions

**Katia Ihadadene:** methodology, validation, visualization, writing – review and editing, formal analysis, writing – original draft, investigation. **Azdah Hamed A Fallatah:** investigation, writing – original draft, methodology, validation, visualization, writing – review and editing, formal analysis. **Yu Zhu:** methodology, visualization, supervision. **Arianna Tolone:** conceptualization, supervision, resources. **François Paquet‐Durand:** conceptualization, writing – review and editing, resources, supervision.

### Peer Review

The peer review history for this article is available at https://www.webofscience.com/api/gateway/wos/peer‐review/10.1111/jnc.70072.

## Supporting information


Figure S1.

Figure S2.

Data S1.

Data S2.

## Data Availability

All data generated or analysed during this study are included in this published article or its Supporting Information.

## References

[jnc70072-bib-0001] Alarautalahti, V. , S. Ragauskas , J. J. Hakkarainen , et al. 2019. “Viability of Mouse Retinal Explant Cultures Assessed by Preservation of Functionality and Morphology.” Investigative Ophthalmology & Visual Science 60, no. 6: 1914–1927. 10.1167/iovs.18-25156. 31042799

[jnc70072-bib-0002] Almasieh, M. , A. M. Wilson , B. Morquette , J. L. Cueva Vargas , and A. Di Polo . 2012. “The Molecular Basis of Retinal Ganglion Cell Death in Glaucoma.” Progress in Retinal and Eye Research 31, no. 2: 152–181. 10.1016/j.preteyeres.2011.11.002. 22155051

[jnc70072-bib-0003] Andrew, P. J. , and B. Mayer . 1999. “Enzymatic Function of Nitric Oxide Synthases.” Cardiovascular Research 43, no. 3: 521–531. 10.1016/s0008-6363(99)00115-7. 10690324

[jnc70072-bib-0004] Baden, T. , P. Berens , K. Franke , M. Román Rosón , M. Bethge , and T. Euler . 2016. “The Functional Diversity of Retinal Ganglion Cells in the Mouse.” Nature 529, no. 7586: 345–350. 10.1038/nature16468. 26735013 PMC4724341

[jnc70072-bib-0005] Baudouin, C. , M. Kolko , S. Melik‐Parsadaniantz , and E. M. Messmer . 2021. “Inflammation in Glaucoma: From the Back to the Front of the Eye, and Beyond.” Progress in Retinal and Eye Research 83: 100916. 10.1016/j.preteyeres.2020.100916. 33075485

[jnc70072-bib-0006] Baydoun, A. R. , R. G. Bogle , J. D. Pearson , and G. E. Mann . 1994. “Discrimination Between Citrulline and Arginine Transport in Activated Murine Macrophages: Inefficient Synthesis of NO From Recycling of Citrulline to Arginine.” British Journal of Pharmacology 112, no. 2: 487–492. 10.1111/j.1476-5381.1994.tb13099.x. 8075867 PMC1910348

[jnc70072-bib-0007] Becker, K. , H. Klein , E. Simon , et al. 2021. “In‐Depth Transcriptomic Analysis of Human Retina Reveals Molecular Mechanisms Underlying Diabetic Retinopathy.” Scientific Reports 11, no. 1: 10494. 10.1038/s41598-021-88698-3. 34006945 PMC8131353

[jnc70072-bib-0008] Belhadj, S. , A. Tolone , G. Christensen , S. Das , Y. Chen , and F. Paquet‐Durand . 2020. “Long‐Term, Serum‐Free Cultivation of Organotypic Mouse Retina Explants With Intact Retinal Pigment Epithelium.” Journal of Visualized Experiments: JoVE 165: 61868. 10.3791/61868. 33311434

[jnc70072-bib-0009] Bicker, G. 2005. “STOP and GO With NO: Nitric Oxide as a Regulator of Cell Motility in Simple Brains.” BioEssays 27, no. 5: 495–505. 10.1002/bies.20221. 15832386

[jnc70072-bib-0010] Blom, J. , T. Giove , M. Deshpande , and W. D. Eldred . 2012. “Characterization of Nitric Oxide Signaling Pathways in the Mouse Retina.” Journal of Comparative Neurology 520, no. 18: 4204–4217. 10.1002/cne.23148. 22592770

[jnc70072-bib-0011] Blom, J. J. , T. A. Blute , and W. D. Eldred . 2009. “Functional Localization of the Nitric Oxide/cGMP Pathway in the Salamander Retina.” Visual Neuroscience 26, no. 3: 275–286. 10.1017/S0952523809990125. 19602301

[jnc70072-bib-0012] Brenes, E. , and A. Gómez . 2016. “Scorpions Maintenance in Captivity for Venom Extraction Purposes in Costa Rica.” Revista de Biología Tropical 64, no. 3: 1019–1027. 10.15517/rbt.v64i3.21138. 29461767

[jnc70072-bib-0013] Bull, N. D. , T. V. Johnson , G. Welsapar , N. W. DeKorver , S. I. Tomarev , and K. R. Martin . 2011. “Use of an Adult Rat Retinal Explant Model for Screening of Potential Retinal Ganglion Cell Neuroprotective Therapies.” Investigative Ophthalmology & Visual Science 52, no. 6: 3309–3320. 10.1167/iovs.10-6873. 21345987 PMC3109030

[jnc70072-bib-0014] Caffé, A. R. , H. Visser , H. G. Jansen , and S. Sanyal . 1989. “Histotypic Differentiation of Neonatal Mouse Retina in Organ Culture.” Current Eye Research 8, no. 10: 1083–1092. 10.3109/02713688908997401. 2612197

[jnc70072-bib-0015] Cantó, A. , T. Olivar , F. J. Romero , and M. Miranda . 2019. “Nitrosative Stress in Retinal Pathologies: Review.” Antioxidants 8, no. 11: 543. 10.3390/antiox8110543. 31717957 PMC6912788

[jnc70072-bib-0016] Carelli, V. , C. La Morgia , and P. Yu‐Wai‐Man . 2023. “Mitochondrial Optic Neuropathies.” Handbook of Clinical Neurology 194: 23–42. 10.1016/B978-0-12-821751-1.00010-5. 36813316

[jnc70072-bib-0017] Cheung, W. S. , I. Bhan , and S. A. Lipton . 2000. “Nitric Oxide (NO) Stabilizes Whereas Nitrosonium (NO+) Enhances Filopodial Outgrowth by Rat Retinal Ganglion Cells In Vitro.” Brain Research 868, no. 1: 1–13. 10.1016/s0006-8993(00)02161-2. 10841882

[jnc70072-bib-0018] Chitranshi, N. , Y. Dheer , M. Abbasi , Y. You , S. L. Graham , and V. Gupta . 2018. “Glaucoma Pathogenesis and Neurotrophins: Focus on the Molecular and Genetic Basis for Therapeutic Prospects.” Current Neuropharmacology 16, no. 7: 1018–1035. 10.2174/1570159X16666180419121247. 29676228 PMC6120108

[jnc70072-bib-0019] Claes, M. , and L. Moons . 2022. “Retinal Ganglion Cells: Global Number, Density and Vulnerability to Glaucomatous Injury in Common Laboratory Mice.” Cells 11, no. 17: 2689. 10.3390/cells11172689. 36078097 PMC9454702

[jnc70072-bib-0020] Costa, K. H. A. , B. D. Gomes , L. C. L. Silveira , et al. 2020. “Ganglion Cells and Displaced Amacrine Cells Density in the Retina of the Collared Peccary (*Pecari tajacu*).” PLoS One 15, no. 10: e0239719. 10.1371/journal.pone.0239719. 33002017 PMC7529232

[jnc70072-bib-0021] Dawson, V. L. , T. M. Dawson , E. D. London , D. S. Bredt , and S. H. Snyder . 1991. “Nitric Oxide Mediates Glutamate Neurotoxicity in Primary Cortical Cultures.” Proceedings of the National Academy of Sciences of the United States of America 88, no. 14: 6368–6371. 10.1073/pnas.88.14.6368. 1648740 PMC52084

[jnc70072-bib-0022] de Vente, J. , H. W. Steinbusch , and J. Schipper . 1987. “A New Approach to Immunocytochemistry of 3',5'‐Cyclic Guanosine Monophosphate: Preparation, Specificity, and Initial Application of a New Antiserum Against Formaldehyde‐Fixed 3',5'‐Cyclic Guanosine Monophosphate.” Neuroscience 22, no. 1: 361–373. 10.1016/0306-4522(87)90226-0. 2819779

[jnc70072-bib-0023] Deppe, L. , A. M. Mueller‐Buehl , T. Tsai , C. Erb , H. B. Dick , and S. C. Joachim . 2024. “Protection Against Oxidative Stress by Coenzyme Q10 in a Porcine Retinal Degeneration Model.” Journal of Personalized Medicine 14, no. 4: 437. 10.3390/jpm14040437. 38673065 PMC11051541

[jnc70072-bib-0024] Dräger, U. C. , and J. F. Olsen . 1981. “Ganglion Cell Distribution in the Retina of the Mouse.” Investigative Ophthalmology & Visual Science 20, no. 3: 285–293. 6162818

[jnc70072-bib-0025] Evangelho, K. , C. A. Mastronardi , and A. de‐la‐Torre . 2019. “Experimental Models of Glaucoma: A Powerful Translational Tool for the Future Development of New Therapies for Glaucoma in Humans‐A Review of the Literature.” Medicina 55, no. 6: 280. 10.3390/medicina55060280. 31212881 PMC6630440

[jnc70072-bib-0026] Fallahian, F. , F. Karami‐Tehrani , S. Salami , and M. Aghaei . 2011. “Cyclic GMP Induced Apoptosis via Protein Kinase G in Oestrogen Receptor‐Positive and ‐Negative Breast Cancer Cell Lines.” FEBS Journal 278, no. 18: 3360–3369. 10.3390/medicina55060280. 21777390

[jnc70072-bib-0027] Farber, D. B. , and R. N. Lolley . 1974. “Cyclic Guanosine Monophosphate: Elevation in Degenerating Photoreceptor Cells of the C3H Mouse Retina.” Science 186, no. 4162: 449–451. 10.1126/science.186.4162.449. 4369896

[jnc70072-bib-0028] Faulkner, I. E. , R. Z. Pajak , M. K. Harte , J. D. Glazier , and R. Hager . 2024. “Voltage‐Gated Potassium Channels as a Potential Therapeutic Target for the Treatment of Neurological and Psychiatric Disorders.” Frontiers in Cellular Neuroscience 18: 1449151. 10.3389/fncel.2024.1449151. 39411003 PMC11473391

[jnc70072-bib-0029] Fiscus, R. R. 2002. “Involvement of Cyclic GMP and Protein Kinase G in the Regulation of Apoptosis and Survival in Neural Cells.” Neurosignals 11, no. 4: 175–190. 10.1159/000065431. 12393944

[jnc70072-bib-0030] Garcia‐Calvo, M. , R. J. Leonard , J. Novick , et al. 1993. “Purification, Characterization, and Biosynthesis of Margatoxin, a Component of Centruroides Margaritatus Venom That Selectively Inhibits Voltage‐Dependent Potassium Channels.” Journal of Biological Chemistry 268, no. 25: 18866–18874. 8360176

[jnc70072-bib-0031] Garthwaite, J. 2005. “Dynamics of Cellular NO‐cGMP Signaling.” Frontiers in Bioscience 10, no. 1‐3: 1868–1880. 10.2741/1666. 15769672

[jnc70072-bib-0032] Gavrieli, Y. , Y. Sherman , and S. A. Ben‐Sasson . 1992. “Identification of Programmed Cell Death In Situ via Specific Labeling of Nuclear DNA Fragmentation.” Journal of Cell Biology 119, no. 3: 493–501. 10.1083/jcb.119.3.493. 1400587 PMC2289665

[jnc70072-bib-0033] Hirooka, K. , D. E. Kourennyi , and S. Barnes . 2000. “Calcium Channel Activation Facilitated by Nitric Oxide in Retinal Ganglion Cells.” Journal of Neurophysiology 83, no. 1: 198–206. 10.1152/jn.2000.83.1.198. 10634867

[jnc70072-bib-0034] Höltje, M. , I. Brunk , J. Grosse , et al. 2007. “Differential Distribution of Voltage‐Gated Potassium Channels Kv 1.1‐Kv1.6 in the Rat Retina During Development.” Journal of Neuroscience Research 85, no. 1: 19–33. 10.1002/jnr.21105. 17075900

[jnc70072-bib-0035] Ignarro, L. J. 1990. “Nitric Oxide. A Novel Signal Transduction Mechanism for Transcellular Communication.” Hypertension 16, no. 5: 477–483. 10.1161/01.hyp.16.5.477. 1977698

[jnc70072-bib-0036] Jayaram, H. , M. Kolko , D. S. Friedman , and G. Gazzard . 2023. “Glaucoma: Now and Beyond.” Lancet 402, no. 10414: 1788–1801. 10.1016/S0140-6736(23)01289-8. 37742700

[jnc70072-bib-0037] Jeon, C. J. , E. Strettoi , and R. H. Masland . 1998. “The Major Cell Populations of the Mouse Retina.” Journal of Neuroscience 18, no. 21: 8936–8946. 10.1016/S0140-6736(23)01289-8. 9786999 PMC6793518

[jnc70072-bib-0038] Judge, S. I. , J. M. Lee , C. T. Bever Jr. , and P. M. Hoffman . 2006. “Voltage‐Gated Potassium Channels in Multiple Sclerosis: Overview and New Implications for Treatment of Central Nervous System Inflammation and Degeneration.” Journal of Rehabilitation Research and Development 43, no. 1: 111–122. 10.1682/JRRD.2004.09.0116. 16847777

[jnc70072-bib-0040] Katsuki, H. , R. Yamamoto , D. Nakata , T. Kume , and A. Akaike . 2004. “Neuronal Nitric Oxide Synthase Is Crucial for Ganglion Cell Death in Rat Retinal Explant Cultures.” Journal of Pharmaceutical Sciences 94, no. 1: 77–80. 10.1254/jphs.94.77. 14745122

[jnc70072-bib-0041] Koeberle, P. D. , and L. C. Schlichter . 2010. “Targeting K(V) Channels Rescues Retinal Ganglion Cells In Vivo Directly and by Reducing Inflammation.” Channels 4, no. 5: 337–346. 10.1038/cdd.2009.113. 20699649 PMC3051871

[jnc70072-bib-0042] Koeberle, P. D. , Y. Wang , and L. C. Schlichter . 2010. “Kv1.1 and Kv1.3 Channels Contribute to the Degeneration of Retinal Ganglion Cells After Optic Nerve Transection In Vivo.” Cell Death and Differentiation 17, no. 1: 134–144. 10.1038/cdd.2009.113. 19696788

[jnc70072-bib-0043] Koriyama, Y. , Y. Takagi , K. Chiba , et al. 2011. “Neuritogenic Activity of a Genipin Derivative in Retinal Ganglion Cells Is Mediated by Retinoic Acid Receptor β Expression Through Nitric Oxide/S‐Nitrosylation Signaling.” Journal of Neurochemistry 119, no. 6: 1232–1242. 10.1111/j.1471-4159.2011.07533.x. 21995424

[jnc70072-bib-0044] Leamey, C. A. , C. L. Ho‐Pao , and M. Sur . 2001. “Disruption of Retinogeniculate Pattern Formation by Inhibition of Soluble Guanylyl Cyclase.” Journal of Neuroscience 21, no. 11: 3871–3880. 10.1523/JNEUROSCI.21-11-03871.2001. 11356875 PMC6762700

[jnc70072-bib-0045] Li, Y. , P. Maher , and D. Schubert . 1997. “Requirement for cGMP in Nerve Cell Death Caused by Glutathione Depletion.” Journal of Cell Biology 139, no. 5: 1317–1324. 10.1083/jcb.139.5.1317. 9382876 PMC2140210

[jnc70072-bib-0046] Lolley, R. N. , D. B. Farber , M. E. Rayborn , and J. G. Hollyfield . 1977. “Cyclic GMP Accumulation Causes Degeneration of Photoreceptor Cells: Simulation of an Inherited Disease.” Science 196, no. 4290: 664–666. 10.1126/science.193183. 193183

[jnc70072-bib-0047] Lozano, A. C. , A. Serrano , D. Salazar , J. V. Rincón , and M. Pardo Bayona . 2024. “Telemedicine for Screening and Follow‐Up of Glaucoma: A Descriptive Study.” Telemedicine Journal and E‐Health 30, no. 7: 1901–1908. 10.1089/tmj.2023.0676. 38662524

[jnc70072-bib-0048] Medeiros, F. A. , R. Lisboa , R. N. Weinreb , J. M. Liebmann , C. Girkin , and L. M. Zangwill . 2013. “Retinal Ganglion Cell Count Estimates Associated With Early Development of Visual Field Defects in Glaucoma.” Journal of Ophthalmology 120, no. 4: 736–744. 10.1016/j.ophtha.2012.09.039. PMC3804164 23246120

[jnc70072-bib-0049] Meyerson, C. , G. Van Stavern , and C. McClelland . 2015. “Leber Hereditary Optic Neuropathy: Current Perspectives.” Clinical Ophthalmology 9: 1165–1176. 10.2147/OPTH.S62021. 26170609 PMC4492634

[jnc70072-bib-0050] Montoliu, C. , M. Llansola , E. Kosenko , R. Corbalán , and V. Felipo . 1999. “Role of Cyclic GMP in Glutamate Neurotoxicity in Primary Cultures of Cerebellar Neurons.” Neuropharmacology 38, no. 12: 1883–1891. 10.2147/OPTH.S62021. 10608283

[jnc70072-bib-0051] Mueller‐Buehl, A. M. , T. Tsai , J. Hurst , et al. 2021. “Reduced Retinal Degeneration in an Oxidative Stress Organ Culture Model Through an iNOS‐Inhibitor.” Biology 10, no. 5: 383. 10.3390/biology10050383. 33925248 PMC8145164

[jnc70072-bib-0052] Munemasa, Y. , and Y. Kitaoka . 2013. “Molecular Mechanisms of Retinal Ganglion Cell Degeneration in Glaucoma and Future Prospects for Cell Body and Axonal Protection.” Frontiers in Cellular Neuroscience 6: 60. 10.3389/fncel.2012.00060. 23316132 PMC3540394

[jnc70072-bib-0053] Neufeld, A. H. 1999. “Nitric Oxide: A Potential Mediator of Retinal Ganglion Cell Damage in Glaucoma.” Survey of Ophthalmology 43, no. 1: S129–S135. 10.1016/s0039-6257(99)00010-7. 10416755

[jnc70072-bib-0054] Nguyen‐Ba‐Charvet, K. T. , and A. Rebsam . 2020. “Neurogenesis and Specification of Retinal Ganglion Cells.” International Journal of Molecular Sciences 21, no. 2: 451. 10.3390/ijms21020451. 31936811 PMC7014133

[jnc70072-bib-0055] Nuschke, A. C. , S. R. Farrell , J. M. Levesque , and B. C. Chauhan . 2015. “Assessment of Retinal Ganglion Cell Damage in Glaucomatous Optic Neuropathy: Axon Transport, Injury and Soma Loss.” Experimental Eye Research 141: 111–124. 10.1016/j.exer.2015.06.006. 26070986

[jnc70072-bib-0056] Oppenheim, R. W. 1985. “Cyclic GMP and Neurone Death.” Nature 313, no. 5999: 248. 10.1038/313248a0. 2982102

[jnc70072-bib-0057] Pang, I. H. , and A. F. Clark . 2007. “Rodent Models for Glaucoma Retinopathy and Optic Neuropathy.” Journal of Glaucoma 16, no. 5: 483–505. 10.1097/IJG.0b013e3181405d4f. 17700292

[jnc70072-bib-0058] Paquet‐Durand, F. , S. M. Hauck , T. van Veen , M. Ueffing , and P. Ekström . 2009. “PKG Activity Causes Photoreceptor Cell Death in Two Retinitis Pigmentosa Models.” Journal of Neurochemistry 108, no. 3: 796–810. 10.1111/j.1471-4159.2008.05822.x. 19187097

[jnc70072-bib-0059] Pereira, I. C. F. , R. van de Wijdeven , H. M. Wyss , H. J. M. Beckers , and J. M. J. den Toonder . 2021. “Conventional Glaucoma Implants and the New MIGS Devices: A Comprehensive Review of Current Options and Future Directions.” Eye 35, no. 12: 3202–3221. 10.1038/s41433-021-01595-x. 34127842 PMC8602385

[jnc70072-bib-0060] Pilz, R. B. , and K. E. Broderick . 2005. “Role of Cyclic GMP in Gene Regulation.” Frontiers in Bioscience 10: 1239–1268. 10.2741/1616. 15769622

[jnc70072-bib-0061] Pivovarov, A. S. , F. Calahorro , and R. J. Walker . 2018. “Na^+^/K^+^‐Pump and Neurotransmitter Membrane Receptors.” Invertebrate Neuroscience 19, no. 1: 1. 10.1007/s10158-018-0221-7. 30488358 PMC6267510

[jnc70072-bib-0062] Power, M. , S. Das , K. Schütze , V. Marigo , P. Ekström , and F. Paquet‐Durand . 2020. “Cellular Mechanisms of Hereditary Photoreceptor Degeneration ‐ Focus on cGMP.” Progress in Retinal and Eye Research 74: 100772. 10.1016/j.preteyeres.2019.07.005. 31374251

[jnc70072-bib-0063] Quadri, M. , A. Comitato , E. Palazzo , et al. 2022. “Activation of cGMP‐Dependent Protein Kinase Restricts Melanoma Growth and Invasion by Interfering With the EGF/EGFR Pathway.” Journal of Investigative Dermatology 142, no. 1: 201–211. 10.1016/j.jid.2021.06.011. 34265328

[jnc70072-bib-0064] Rathnasamy, G. , V. Sivakumar , P. Rangarajan , W. S. Foulds , E. A. Ling , and C. Kaur . 2014. “NF‐κB‐Mediated Nitric Oxide Production and Activation of Caspase‐3 Cause Retinal Ganglion Cell Death in the Hypoxic Neonatal Retina.” Investigative Ophthalmology & Visual Science 55, no. 9: 5878–5889. 10.1167/iovs.13-13718. 25139733

[jnc70072-bib-0065] Roy, A. , A. Tolone , R. Hilhorst , J. Groten , T. Tomar , and F. Paquet‐Durand . 2022. “Kinase Activity Profiling Identifies Putative Downstream Targets of cGMP/PKG Signaling in Inherited Retinal Neurodegeneration.” Cell Death Discovery 8, no. 1: 93. 10.1038/s41420-022-00897-7. 35241647 PMC8894370

[jnc70072-bib-0066] Sandner, P. , D. P. Zimmer , G. T. Milne , M. Follmann , A. Hobbs , and J. P. Stasch . 2021. “Soluble Guanylate Cyclase Stimulators and Activators.” Handbook of Experimental Pharmacology 264: 355–394. 10.1007/164_2018_197. 30689085

[jnc70072-bib-0067] Schnichels, S. , F. Paquet‐Durand , M. Löscher , et al. 2021. “Retina in a Dish: Cell Cultures, Retinal Explants and Animal Models for Common Diseases of the Retina.” Progress in Retinal and Eye Research 81: 100880. 10.1016/j.preteyeres.2020.100880. 32721458

[jnc70072-bib-0068] Taimor, G. , B. Hofstaetter , and H. M. Piper . 2000. “Apoptosis Induction by Nitric Oxide in Adult Cardiomyocytes via cGMP‐Signaling and Its Impairment After Simulated Ischemia.” Cardiovascular Research 45, no. 3: 588–594. 10.1016/s0008-6363(99)00272-2. 10728380

[jnc70072-bib-0069] Tolone, A. , W. Haq , A. Fachinger , et al. 2023. “The PKG Inhibitor CN238 Affords Functional Protection of Photoreceptors and Ganglion Cells Against Retinal Degeneration.” International Journal of Molecular Sciences 24, no. 20: 15277. 10.3390/ijms242015277. 37894958 PMC10607377

[jnc70072-bib-0070] Tsai, T. I. , B. v. Bui , and A. J. Vingrys . 2009. “Dimethyl Sulphoxide Dose‐Response on Rat Retinal Function.” Documenta Ophthalmologica. Proceedings Series 119, no. 3: 199–207. 10.1007/s10633-009-9191-8. 19763650

[jnc70072-bib-0071] Valdés, J. , L. Trachsel‐Moncho , A. Sahaboglu , et al. 2016. “Organotypic Retinal Explant Cultures as In Vitro Alternative for Diabetic Retinopathy Studies.” ALTEX 33, no. 4: 459–464. 10.14573/altex.1603111. 27159027

[jnc70072-bib-0072] Vorwerk, C. K. , B. T. Hyman , J. W. Miller , et al. 1997. “The Role of Neuronal and Endothelial Nitric Oxide Synthase in Retinal Excitotoxicity.” Investigative Ophthalmology & Visual Science 38, no. 10: 2038–2044. 9331267

[jnc70072-bib-0073] Wang, X. , and P. J. Robinson . 1997. “Cyclic GMP‐Dependent Protein Kinase and Cellular Signaling in the Nervous System.” Journal of Neurochemistry 68, no. 2: 443–456. 10.1046/j.1471-4159.1997.68020443.x. 9003029

[jnc70072-bib-0074] Xu, J. , L. Morris , A. Thapa , et al. 2013. “cGMP Accumulation Causes Photoreceptor Degeneration in CNG Channel Deficiency: Evidence of cGMP Cytotoxicity Independently of Enhanced CNG Channel Function.” Journal of Neuroscience 33, no. 37: 14939–14948. 10.1523/JNEUROSCI.0909-13.2013. 24027293 PMC3771030

[jnc70072-bib-0075] Yang, F. , H. Ma , M. R. Butler , and X. Q. Ding . 2020. “Potential Contribution of Ryanodine Receptor 2 Upregulation to cGMP/PKG Signaling‐Induced Cone Degeneration in Cyclic Nucleotide‐Gated Channel Deficiency.” FASEB Journal 34, no. 5: 6335–6350. 10.1096/fj.201901951RR. 32173907 PMC7299158

[jnc70072-bib-0076] Zhong, Y. S. , J. Wang , W. M. Liu , and Y. H. Zhu . 2013. “Potassium Ion Channels in Retinal Ganglion Cells (Review).” Molecular Medicine Reports 8, no. 2: 311–319. 10.3892/mmr.2013.1508. 23732984

