# Supplementary figures and images for: Inhibition of cGMP‐Signalling Rescues Retinal Ganglion Cells From Axotomy‐Induced Degeneration
Source: J Neurochem. 2025 Apr 24;169(4):e70072. doi: 10.1111/jnc.70072 (PMC12019586; doi:10.1111/jnc.70072)

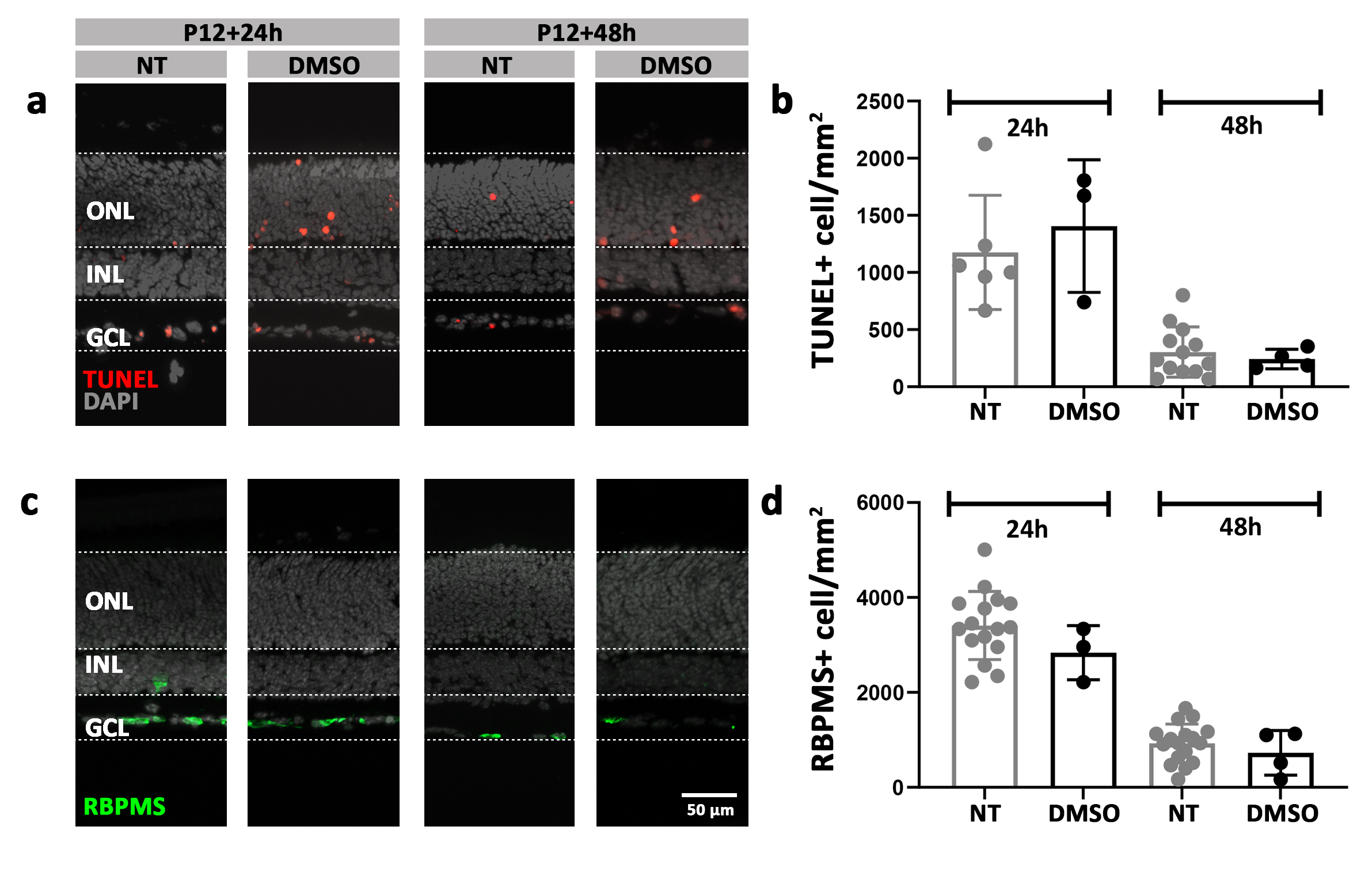

Supplement: Supplementary file 1 — Figure S1. [file JNC-169-0-s003.tif]

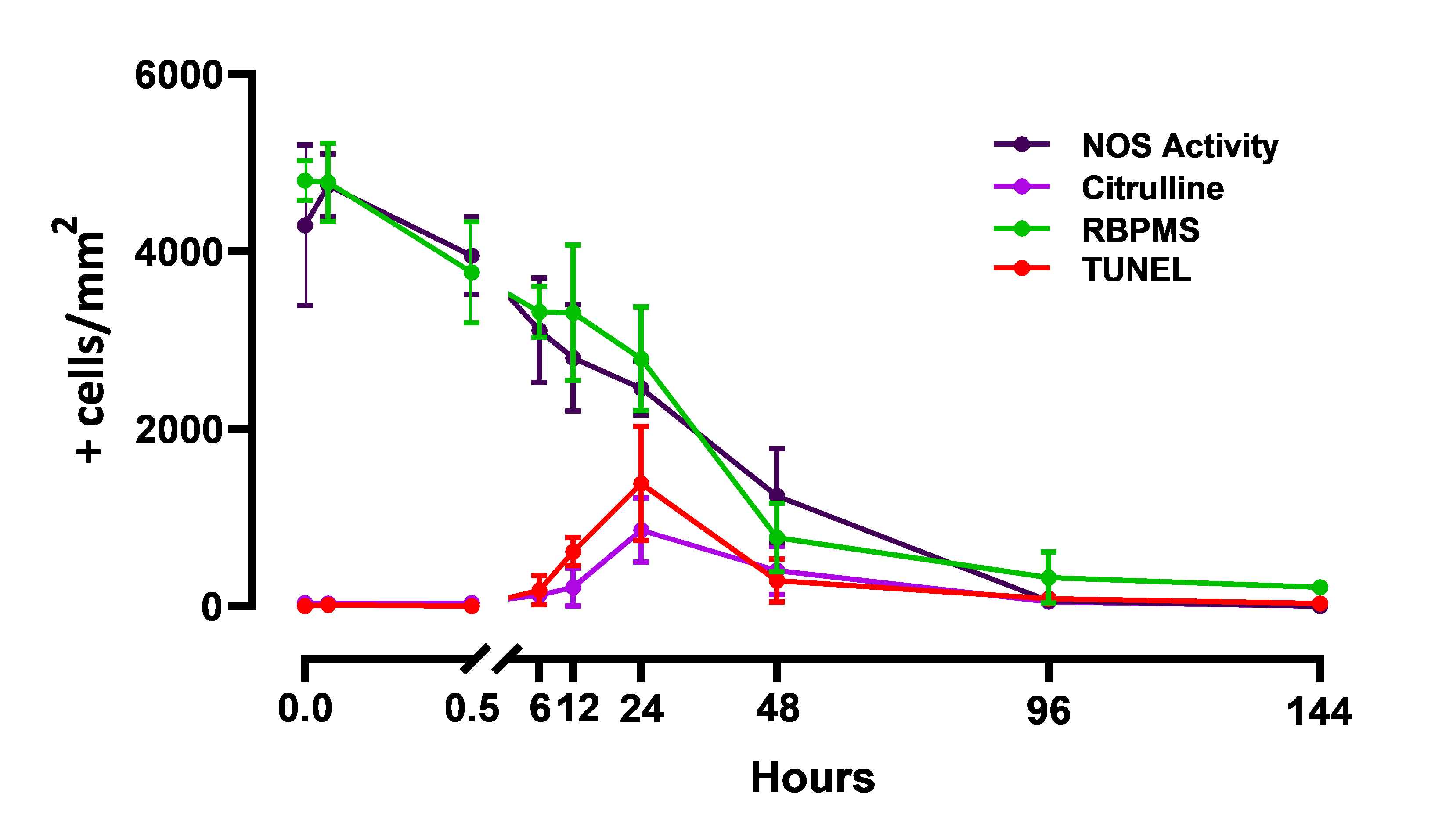

Supplement: Supplementary file 2 — Figure S2. [file JNC-169-0-s002.tif]
