# Supplementary material for: Inhibition of cGMP‐Signalling Rescues Retinal Ganglion Cells From Axotomy‐Induced Degeneration
Source: J Neurochem. 2025 Apr 24;169(4):e70072. doi: 10.1111/jnc.70072 (PMC12019586; doi:10.1111/jnc.70072)
Supplement: Supplementary file 3 — Data S1. [file JNC-169-0-s001.pdf]

Inhibition of cGMP-signalling rescues retinal ganglion cells from axotomy-induced degeneration

Authors:

Katia Ihadadene [1,2*], Azdah Hamed A Fallatah [2,3,4*], Yu Zhu [2,4], Arianna Tolone [2], François Paquet-Durand [2]

Affiliations:

1 Graduate School INTHERAPI, Burgundy University, Dijon, France

2 Institute for Ophthalmic Research, University of Tübingen, Tübingen, Germany

3 Graduate School for Molecular Medicine, University of Tübingen, Tübingen, Germany

4 Graduate School for Cellular and Molecular Neuroscience, University of Tübingen, Tübingen, Germany

* These authors should be considered as equal first authors.

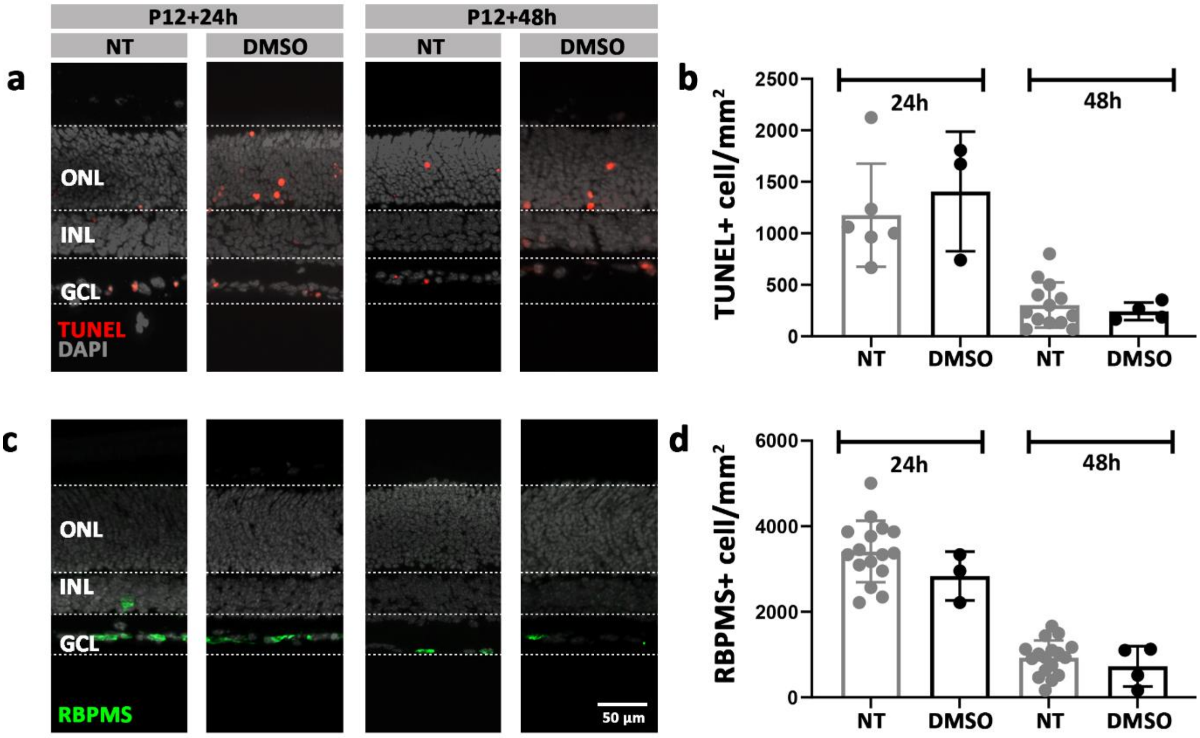

Supplemental Figure 1. Low DMSO concentrations have no effect on RGC viability. Retinal explant cultures derived from wild-type (WT) mice at post-natal day 12 were cultivated for 24 h and 48 h either with complete medium (CM) (NT condition) or with CM containing 0.1 % DMSO. **a**, **b**) TUNEL assay (red) and quantification of TUNEL positive cells within the ganglion cell layer (GCL). **c**, **d**) RBPMS-immunostaining (green) and counts of RBPMS-positive retinal ganglion cells (RGCs). Quantifications indicate positive cells/mm^2; data points in bar graphs represent retinal explants from different animals; testing was performed on n = 5-17 animals. Error bars: Mean with SD; 0.1 % DMSO was compared to NT using the non-parametric Mann-Whitney U test. ONL = outer nuclear layer, INL = inner nuclear layer; scale bar: 50 µm.

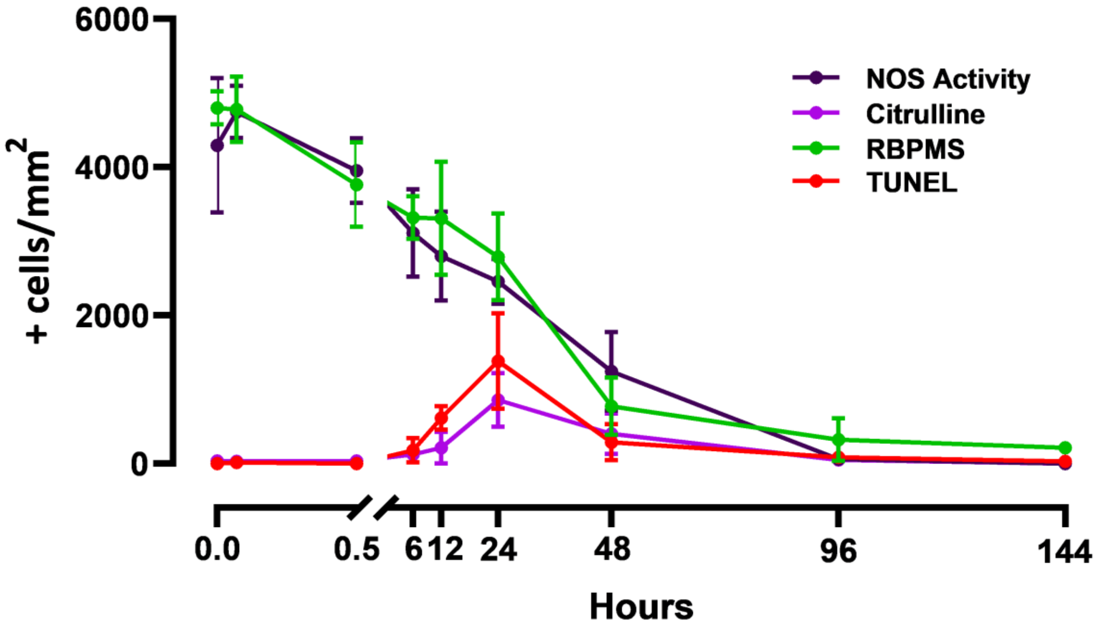

Supplemental Figure 2. Wild-type retina was explanted at post-natal day (P) 12 and cultured for periods ranging from 0 hours to 6 days *in vitro.* Remarkably, NOS activity (dark purple) was already high at the first analysis time-point after optic nerve transection and decreased gradually over time. RBPMS immunostaining (green) detected retinal ganglion cells (RGCs). Their numbers steadily decreased after axotomy. TUNEL assay (red) detected dying RGCs. Cell death peaked at 24 h after optic nerve transection, followed by a decrease in TUNEL-positive cells and Citrulline-positive cells until 6 days. Quantifications indicate positive cells/mm^2. Statistical testing was performed on n = 3 to 5 different retinae from different animals.
